# Fluorescent hexaaryl- and hexa-heteroaryl[3]radialenes: Synthesis, structures, and properties

**DOI:** 10.3762/bjoc.8.7

**Published:** 2012-01-11

**Authors:** Antonio Avellaneda, Courtney A Hollis, Xin He, Christopher J Sumby

**Affiliations:** 1 School of Chemistry & Physics, The University of Adelaide, Adelaide, SA 5005, Australia. Phone: +61 8 8303 7406. Fax: +61 8 8303 4358

**Keywords:** anion–π interactions, cross-conjugated compounds, electron deficient compounds, fluorescence, radialenes

## Abstract

The syntheses of three new [3]radialenes – hexakis(3,5-dimethylpyrazolyl)-, hexakis(3-cyanophenyl)-, and hexakis(3,4-dicyanophenyl)[3]radialene (**1**–**3**) – are reported. Compound **3** is obtained in five steps with an excellent yield of 76% in the key step. Compared to that, the respective steps of the syntheses of **1** and **2** result in lower yields. All compounds adopt a double bladed propeller conformation in solution. Compound **3** is considerably more electron deficient than previously reported hexaaryl[3]radialenes, with reduction potentials of −0.06 and −0.45 V in CH_2_Cl_2_. The compounds mostly display red fluorescence with large Stokes shifts.

## Introduction

**Figure 1:**
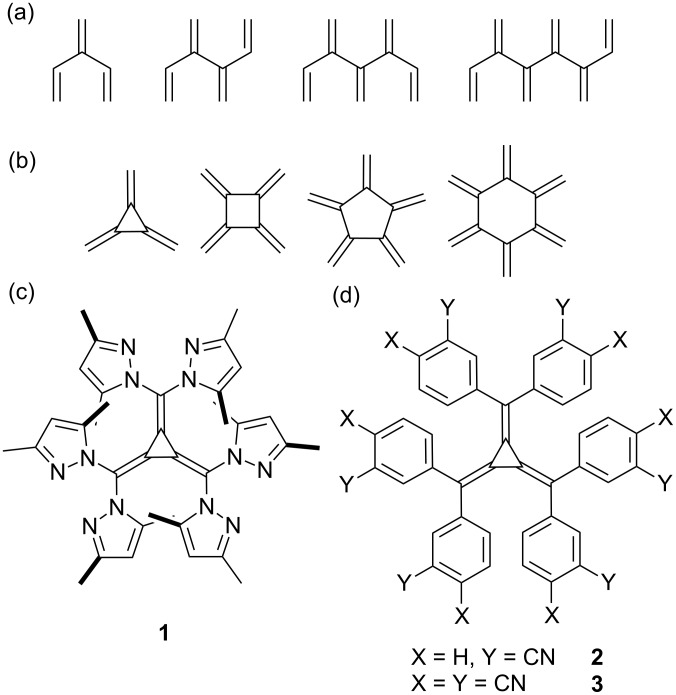
The structures of a) the parent [3]-, [4]-, [5]-, and [6]dendralenes and b) the corresponding radialenes. The structure of c) hexakis(3,5-dimethylpyrazolyl)[3]radialene (**1**) and d) hexakis(3-cyanophenyl)[3]radialene (**2**) and hexakis(3,4-dicyanophenyl)[3]radialene (**3**).

Cross-conjugated compounds are those which “contain three unsaturated groups, two of which though conjugated to a third unsaturated centre are not conjugated to each other” [1–2]. Such compounds display interesting physical properties and chemical reactivity [1–2]. The parent dendralenes are acyclic cross-conjugated polyenes (Figure 1a) whose relative instability had prevented them from being successfully produced in large quantities [3]. Recently, Sherburn reported the synthesis of the first six members of the parent dendralene series on gram scales [4–8]. Initial studies have shown that the odd-numbered dendralenes are much more chemically reactive than the even-numbered dendralenes.

In turn, radialenes are cyclic cross-conjugated polyenes which exhibit a general formula of C_2_*_n_*H_2_*_n_* and contain *n* ring atoms and *n* exocyclic double bonds (Figure 1b) [9]. Like their acyclic dendralene analogues, radialenes have proven to be a significant synthetic challenge. The parent compounds [3]- and [4]radialene were initially prepared in the 1960’s, however, progress since then has been protracted [10–12]. Derivatives of [3]radialene containing aryl and heteroaryl moieties have gained more interest than the parent compound itself due to their increased stability. The preparation of hexaaryl[3]radialenes, using Fukunaga’s method of reacting stabilised carbanions with tetrachlorocyclopropene [13–14], was originally reported by Oda [15–16]. Hexapyridyl[3]radialenes were obtained shortly afterwards with the synthesis of hexa(2-pyridyl)[3]radialene being reported concurrently by Oda and Steel [17–18].

The coordination chemistry of hexaaryl- and hexapyridyl[3]radialenes has been studied to a limited extent [18–21]. Three coordination modes were observed for hexa(2-pyridyl)[3]radialene with Ag(I): A discrete M_6_L_2_ cage, a 1-D coordination polymer composed of M_3_L_2_ cages bridged by linear silver atoms, and a second 1-D coordination polymer where the radialene ligand acts as a tetradentate bridge [18–19]. Hexa(4-pyridyl)[3]radialene also acts as a bridging ligand in a 3-D coordination polymer with AgClO_4_ [20], while isomorphous 6,3-connected 2-D coordination polymers were obtained when hexakis(4-cyanophenyl)[3]radialene was reacted with AgPF_6_ and AgClO_4_ [21].

Anion–π interactions have recently received much attention [22–26], where the π-system is typically an electron deficient heterocyclic system. The electron deficient nature of the hexaaryl[3]radialenes is borne out in structures of these compounds which also show anion–π interactions and CH···X_anion_ hydrogen bonding in the solid-state [17,21]. Hexa(2-pyridyl)[3]radialene exhibits a very short contact (2.67 Å) between the radialene π-system and a fluoride anion in its hexanuclear silver(I) cage structure [18]. Hexakis(4-cyanophenyl)[3]radialene also shows anion–π interactions involving the PF_6_^−^ and ClO_4_^−^ anions in the solid-state [21]. Cyclic voltammetry experiments show that the most electron deficient hexaaryl[3]radialene synthesised thus far is hexakis(4-cyanophenyl)[3]radialene [17].

The focus of our current work is the synthesis of new hexaaryl- and hexa-heteroaryl[3]radialenes which are more electron deficient and, therefore, more disposed toward forming anion–π interactions. Ultimately, the most desirable of these compounds will be able to form capsular metallo-supramolecular assemblies. Herein, we outline the synthesis of three new [3]radialene derivatives (**1**–**3**), including the most electron deficient hexaaryl[3]radialene observed thus far, and studies of their electrochemical and photophysical properties.

## Results and Discussion

The first new [3]radialene compound targeted was hexa(pyrazol-1-yl)[3]radialene. However, the required starting material, 1-[(1*H*-pyrazol-1-yl)methyl]-1*H*-pyrazole, undergoes non-specific lithiation with *n-*butyllithium [27]. As selective lithiation at the methane position is required, bis(3,5-dimethylpyrazol-1-yl)methane [28] – where the 3- and 5-positions are blocked with methyl groups – was used to give hexakis(3,5-dimethylpyrazol-1-yl)[3]radialene (**1**) via Fukunaga’s method in 14% yield. The poor yield for this reaction was attributed to the considerable steric hindrance deriving from the 12 methyl substituents and, despite considerable effort, could not be optimised further. The compound was readily identified by its intense yellow–green solutions and the considerable upfield shifts of the pyrazole H4 proton (5.69 ppm) and the methyl hydrogen atoms (1.80 and 2.07 ppm) arising from the double-bladed propeller conformation adopted by **1**.

**Scheme 1:**
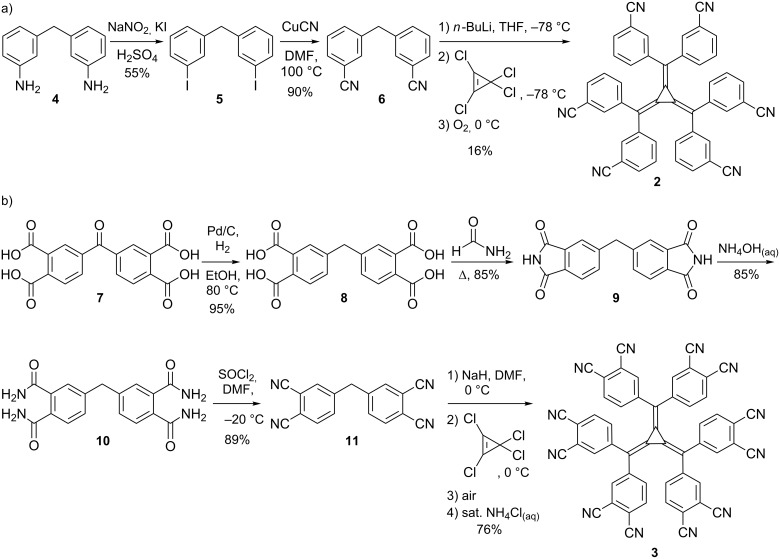
Synthesis of (a) hexakis(3-cyanophenyl)[3]radialene (**2**) and (b) hexakis(3,4-dicyanophenyl)[3]radialene (**3**).

In addition to the study of [3]radialenes with heterocyclic donors [18–19], we have devoted substantial attention to the study of [3]radialenes with nitrile donors in the 4-position of the aryl ring [21] and thus, undertook the synthesis of the isomeric compound, hexakis(3-cyanophenyl)[3]radialene (**2**). The precursor **6** was synthesised from 3,3’-diaminodiphenylmethane (**4**) [29–31] in two steps: diazotisation and reaction with potassium iodide to afford 3,3’-diiododiphenylmethane (**5**) [32] followed by treatment with copper cyanide to give 3,3’-dicyanodiphenylmethane (**6**) rather than the literature route via diphenylmethane-3,3’-dicarboxamide [33]. [3]Radialene **2** was obtained from **6** in 16% yield (Scheme 1a). The marked difference in yields between **2** and hexakis(4-cyanophenyl)[3]radialene, 73% reported by Oda [15], is a consequence of the relative stability of the carbanions of the two dicyanodiphenylmethane precursors.

To maintain a high yielding synthesis whilst investigating the substitution in the 3-position, a new strategy was developed which involved the retention of an electron withdrawing group in the 4-position while incorporating new substitutents at the 3-position. The simplest demonstration of this approach is the preparation of hexakis(3,4-dicyanophenyl)[3]radialene (**3**), which was synthesised in excellent yield (76%, Scheme 1b). The precursor methane **11** was obtained from **7** in four steps: hydrogenation on 5% Pd/C to **8** [34], followed by reaction with formamide to give the corresponding diimide **9**, treatment with aqueous ammonia solution to yield the tetraamide **10** and finally dehydration to the tetranitrile **11** using thionyl chloride [35–36]. Initial attempts to synthesise **3** using the standard procedure [15–16] were unsuccessful. In fact, the far greater acidity of **11** (compared to **6** or 4,4’-dicyanodiphenylmethane) allowed the use of sodium hydride instead of *n*-butyllithium as a base in the synthesis of **3**. Following aerial oxidation of the dianion, **3** was isolated by precipitation in saturated ammonium chloride solution.

**Figure 2:**
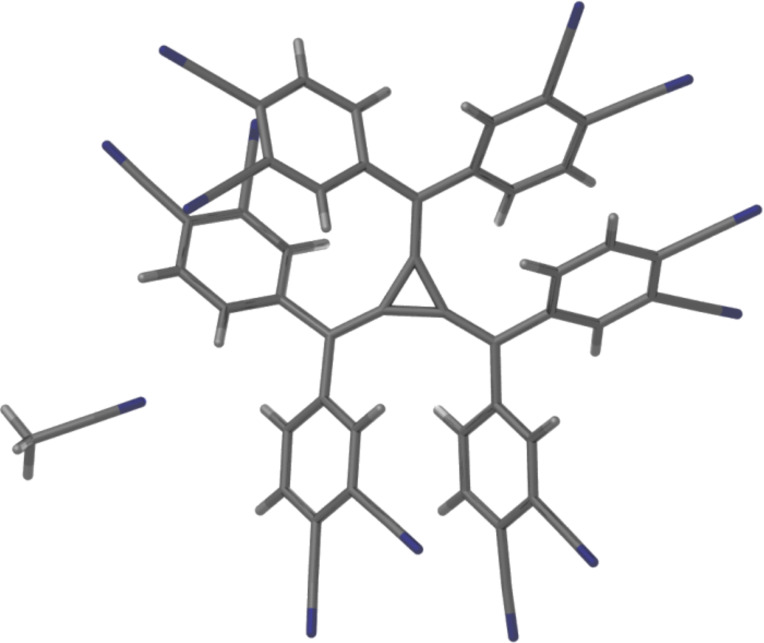
A perspective view of the asymmetric unit of **3**.

Small red crystals of compound **3** [37], that are suitable for X-ray crystallography, were obtained by slow evaporation in acetonitrile. The ligand crystallises in the monoclinic space group *P*2_1_/*c* and the asymmetric unit contains one molecule of the radialene ligand and an acetonitrile solvate molecule (Figure 2). The small crystals were weakly diffracting but the structure refined to *R*_1_ 6.82% with no significant disorder problems. As expected, the [3]radialene core is planar and the “arms” extend in a double-bladed propeller conformation with torsion angles of ca. 39° on average which is common for hexaaryl[3]radialenes [18–20]. The nitrile substituents in the 4-position extend directly out from the structure in approximately the same plane as the core, whereas those in the 3-position are situated above and below the plane. In the conformation observed, four are up and two are directed down. This type of conformation is less commonly encountered as symmetrical arrangements of multi-armed compounds, those with no net dipole, are more favoured [38]. Bond lengths and angles about the central core are consistent with a [3]radialene derivative. Each [3]radialene forms three lone pair-nitrile to electron deficient [3]radialene core carbon contacts [22–26] within the structure, with distances of 3.205, 3.219 and 3.267 Å.

[3]Radialene compounds have been shown to undergo two one-electron reductions to yield the radical anion and dianion species [1]. In this context, in materials science [1,39–40], there is considerable interest in the use of the carbanions obtained by reduction. Up to now, the most electron deficient hexaaryl[3]radialene reported, hexakis(4-cyanophenyl)[3]radialene, recorded reduction potentials of −0.63 and −1.03 V in dichloromethane (Table 1) [17]. Isomer **2** is slightly harder to reduce than hexakis(4-cyanophenyl)[3]radialene, with potentials of −0.80 and −1.32 V in dichloromethane. This is due to the inability of the nitrile groups in the 3-position to stabilise the anion by resonance delocalisation. A similar effect on the reduction potentials is observed for the pyridyl series [18,20,41], whereby the change from a 2-substituted pyridine to a 3-substituted azine ring system results in a compound that is 0.10 V more difficult to reduce. Conversely, compound **3** is very easy to reduce. This is shown by its reduction potentials of −0.06 and −0.45 V, respectively, and is attributed to the combined electron-withdrawing effect of the twelve nitrile substituents. While compound **3** is considerably more electron deficient than other hexaaryl[3]radialenes, it still exists in its neutral form. In contrast, other related [3]radialene compounds – such as hexacyano[3]radialene [13] – exist as stable dianions. Among the [3]radialene compounds we have been studying, this attributes a ‘goldilocks’-type electron deficiency to compound **3** making it suitable for investigating anion–π interactions. The compound is considerably electron deficient, however, it is still maintained in its neutral form. This will allow us to further study anion–π interactions with these systems [21]. The opposite extreme exists for compound **1** because the electron rich azole rings and methyl substituents make it particularly hard to reduce. Its first reduction potential is −1.21 V and its second was not observed as it occurs outside the solvent window for dichloromethane.

**Table 1:** Electrochemical potentials for the synthesised [3]radialenes **1**–**3** and related compounds.

compound – [3]radialene	*E*_(1)_	*E*_(2)_	reference

**1**	−1.21^a,b^	—	this work
hexakis(4-bromophenyl)-	−1.29^c^	−1.77^c^	[17]
hexakis(4-carbomethoxyphenyl)-	−1.03^c^	−1.33^c^	[17]
hexa(2-pyridyl)-	−0.93^a,b^	−1.29^a,b^	[41]
	−1.15^c^	−1.55^c^	[17]
hexa(3-pyridyl)-	−1.03^a,b^	−1.48^a,b,d^	[41]
	−1.17^c^	−1.64^c^	[17]
hexa(4-pyridyl)-	−1.02^a,b^	−1.33^a,b,d^	[41]
**2**	−0.80^a,b^	−1.32^a,b,d^	this work
hexakis(4-cyanophenyl)-	−0.63^a,b^	−1.03^a,b^	this work
	−0.86^d^	−1.11^d^	[17]
**3**	−0.06^a,b^	−0.45^a,b^	this work
hexacyano-	+1.13^e^	+0.34^e^	[13]

^a^Potentials (V) measured in CH_2_Cl_2_/0.1 mol·L^−1^ [(*n*-C_4_H_9_)_4_]NPF_6_ (the ferrocene/ferrocenium couple occurred at +0.46 V vs Ag/Ag^+^). ^b^Uncertainty in *E*_1/2_ values ca*.* ± 0.02 V. ^c^Potentials (V) measured in DMF/0.1 mol·L^−1^ [(*n*-C_4_H_9_)_4_]NClO_4_ (the ferrocene/ferrocenium couple occurred at +0.16 V vs Ag/Ag^+^). ^d^Irreversible (approximate value estimated from anodic half-scan). ^e^Potentials (V) measured in CH_3_CN/0.1 mol·L^−1^ (various electrodes and supporting electrolytes used).

**Figure 3:**
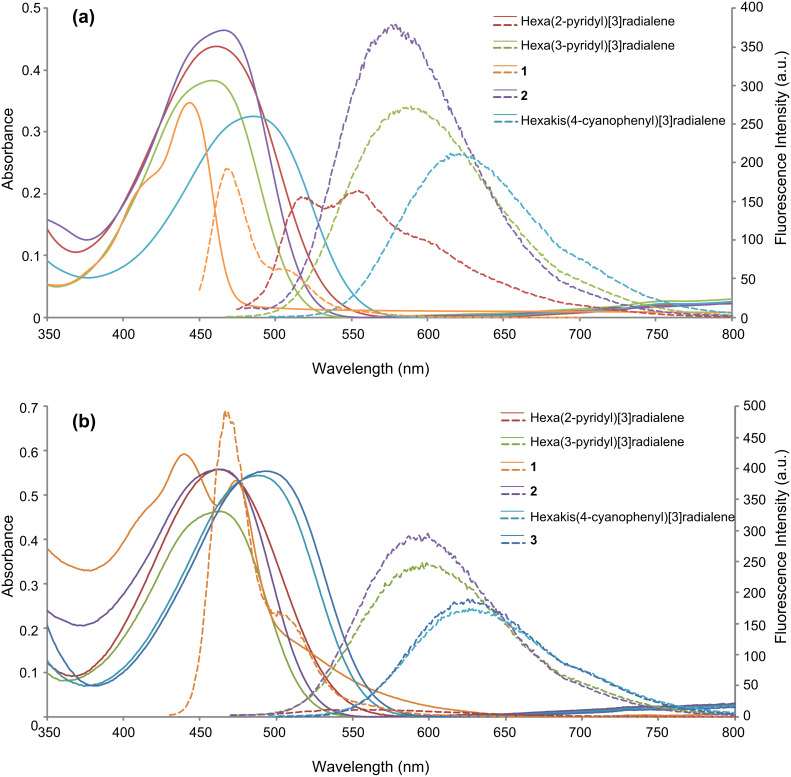
(a) UV–visible (bold line) and fluorescence (dashed line) spectra of **1**, **2**, hexa(2-pyridyl)[3]radialene, hexa(3-pyridyl)[3]radialene, and hexakis(4-cyanophenyl)[3]radialene in dichloromethane. (b) UV–visible (bold line) and fluorescence (dashed line) spectra of **1**, **2**, **3**, hexa(2-pyridyl)[3]radialene, hexa(3-pyridyl)[3]radialene, and hexakis(4-cyanophenyl)[3]radialene in acetone.

Most hexaaryl[3]radialenes are orange or red in colour with UV–visible absorption maxima in the range of 460–490 nm in dichloromethane (Table 2, Figure 3a), with **2** and **3** being consistent with this observation. Compared to that, compound **1** is a yellow–brown solid with a λ_max_ of 443 nm which dissolves to produce yellow–green solutions. In dichloromethane the absorbance maxima for **1** is around 443 nm (logε = 4.33), with a shoulder around 415 nm. In contrast to that, the UV–visible spectra in acetone shows two absorption peaks at around 440 and 475 nm, as well as a broad tail that extends out to approximately 600 nm. The fact that the concentrations of both solutions of **1** are similar (~0.04 mM) indicates that **1** – which has 12 methyl substituents arranged above and below the [3]radialene core – aggregates in the more polar acetone solvent (polarity index for dichloromethane 3.1; acetone 5.1). The less hydrophobic [3]radialenes, **2** and **3**, do not show the same behaviour. The more electron deficient derivatives, **2** and **3**, along with the previously synthesised compounds hexa(2-pyridyl)[3]radialene, hexa(3-pyridyl)[3]radialene, and hexakis(4-cyanophenyl)[3]radialene, show a weak absorption in the near infrared (around 800 nm). This is consistent with spectra observed for the radical anions [17].

**Table 2:** Visible absorption maxima, molar extinction coefficient ε and fluorescence emission maxima for various hexaaryl[3]radialene compounds.

compound – [3]radialene	λ_max_ (nm)	logε	fluorescence max (nm)^a^	reference

hexa(2-pyridyl)-	464^a^	4.53	555^a^	this work
	463^b^	4.25	564^b^	this work
hexa(3-pyridyl)-	465^a^	4.48	585^a^	this work
	464^b^	4.16	598^b^	this work
hexa(4-pyridyl)-	463^a^	4.62	not reported	[41]
**1**	443^a^	4.33	467^a^	this work
	440^b^	4.13	468^b^	this work
**2**	461^a^	4.21	576^a^	this work
	461^b^	4.24	595^b^	this work
hexakis(4-cyanophenyl)-	489^a^	4.42	620^a^	this work
	487^b^	4.37	626^b^	this work
**3**	493^b^	4.39	625^b^	this work
hexaphenyl-	467^a^	4.42	617^a^	[42]
hexakis(4-chlorophenyl)-	483^a^	4.68	606^a^	[42]
hexakis(4-bromophenyl)-	485^a^	4.64	not reported	[15]
hexakis(4-iodophenyl)-	492^a^	4.73	not reported	[15]

^a^UV–visible and fluorescence spectra measured in dichloromethane. ^b^UV–visible and fluorescence spectra measured in acetone.

The fluorescence of hexaaryl[3]radialenes has not been widely studied [42]. Solutions of compound **1** visibly fluoresce bright blue; its fluorescence maximum being 467 nm in dichloromethane with a Stokes shift of 124 nm (Table 2). The absorption and fluorescence properties of compound **3** had to be measured in acetone due to its limited solubility in dichloromethane (Figure 3). Hexakis(4-cyanophenyl)[3]radialene and compounds **2** and **3** also exhibit large Stokes shifts, of at least 130 nm, for their fluorescence maxima in acetone. The large Stokes shifts are consistent with the HOMO of the [3]radialene being located predominantly on the exocyclic double bonds [40]. As a consequence, the electronic structure of the excited state is considerably more polar than that of the ground state.[42] This is consistent with the Stokes shift being 10–20 nm larger for the spectra measured in acetone and with calculations that show intramolecular charge transfer character in the lowest excited states for hexaaryl[3]radialene derivatives [42]. In the excited state, rotation about the exocyclic bonds of the cyclopropane ring is also possible. The fluorescence emission of the hexaaryl[3]radialenes, coupled with the large Stokes shifts, suggests that these compounds would be useful as sensor components. Indeed, our efforts to investigate anion–π interactions [43] are being undertaken with a view to utilise [3]radialenes as a building block for the synthesis of anion sensors.

## Conclusion

In summary, this work has shown that the range of the available [3]radialene derivatives can be extended. However, these extensions are limited depending on the nature and position of the substituents. It is necessary to maintain an electron-withdrawing group in the 4-position of the aryl ring and to limit the steric bulk about the core. As the precursor methane forms a very stable carbanion and the substituents provide minimal steric hindrance, compound **3** is obtained with an excellent yield of 76% in the key step. In contrast to that, the final steps of the syntheses of **1** and **2** are more difficult because the precursors have greater steric bulk and do not form carbanions with the requisite stability, respectively. In this regard, precursor active methylene compounds with functional groups that are able to coordinate transition metals in the 3-position and a nitro group *para* to the methylene are under investigation in our laboratory.

Compounds **2** and **3** are electron deficient, **3** noticeably so, and undergo facile reductions to their radical anion and dianion species. We expect that the extension of these compounds in the 3-position will lead to the formation of new cage structures [18] which will utilise the electron deficient nature of the [3]radialene core to act as anion receptors. The useful electrochemical and fluorescence properties of these compounds may allow them to be employed as building blocks of anion sensors.

## Experimental

### General experimental

Melting points were determined using a Gallenkamp variable heat melting point apparatus and are uncorrected. UV–visible absorption spectra were recorded on a Varian CARY 5000 spectrophotometer. Samples were dissolved in dichloromethane or acetone at a concentration of approximately 0.03 mM. Fluorescence spectra were recorded on a Varian CARY eclipse spectrophotometer. Samples were dissolved in dichloromethane or acetone at a concentration of approximately 0.01 mM. Infrared spectra were recorded using a Perkin Elmer Spectrum 100 FTIR spectrometer with universal ATR sampling accessory. The Campbell microanalytical laboratory at the University of Otago performed the elemental analyses.

Low resolution electrospray ionisation mass spectra (ESIMS) were recorded on a Finnigan LCQ mass spectrometer. Samples were dissolved in HPLC grade methanol or acetonitrile with a concentration of 0.01 mg/cm^3^. High resolution electrospray ionisation mass spectroscopy (ESI-HRMS) was performed by the Adelaide Proteomics Centre using an LTQ Orbitrap XL ETD spectrometer. ^1^H NMR spectra were recorded on a Varian Gemini 300 MHz spectrometer (75 MHz for ^13^C NMR) or a Varian Inova 600 MHz spectrometer (150 MHz for ^13^C NMR). ^1^H NMR spectra recorded in CDCl_3_ were referenced to the internal standard Me_4_Si (0 ppm). ^1^H NMR spectra recorded in DMSO-*d*_6_ and acetone-*d*_6_ were referenced to the solvent peaks 2.50 ppm and 2.05 ppm, respectively. ^13^C NMR spectra recorded in CDCl_3_, DMSO-*d*_6_ and acetone-*d*_6_ were referenced to the solvent peaks 77.0 ppm, 39.5 ppm and 29.9 ppm, respectively. Unless otherwise stated, reagents were obtained from commercial sources and used as received. Bis(3,5-dimethylpyrazol-1-yl)methane [28] and 3,3’-diaminodiphenylmethane [29–31] were synthesised according to literature procedures. Solvents were dried by literature procedures [44] and freshly distilled as required.

### Cyclic voltammetry

Cyclic voltammetry measurements were performed on a PAR Model 263A potentiostat under nitrogen. Measurements were recorded on 1 mM solutions in dichloromethane/0.1 M [(*n*-C_4_H_9_)_4_]NPF_6_] solution using a platinum working electrode, platinum wire auxiliary and pseudo-reference electrodes. Ferrocene was added as an internal standard on completion of each experiment and tabulated potentials are given vs the saturated calomel electrode [E_0_(Fc/Fc^+^) = 460 mV vs SCE (dichloromethane)]. Cyclic voltammetry was performed with a sweep rate of 100 mVs^−1^.

#### X-ray crystallography

Crystals were mounted under oil on a loop and X-ray diffraction data were collected at 150(2) K with Mo Kα radiation (λ = 0.71073 Å) using an Oxford Diffraction X-Calibur Diffractometer fitted with an Eos CCD detector. The data set was corrected for absorption using a multi-scan method. Structures were solved by direct methods using SHELXS-97 [45] and refined by full-matrix least squares on *F*2 by SHELXL-97 [46], interfaced through the program X-Seed [47]. In general, all non-hydrogen atoms were refined anisotropically and hydrogen atoms were included as invariants at geometrically estimated positions. CCDC 824692 contains the supplementary crystallographic data for this structure. These data can be obtained free of charge from The Cambridge Crystallographic Data Centre via http://www.ccdc.cam.ac.uk/data_request/cif.

#### Synthetic procedures

**Hexakis(3,5-dimethylpyrazolyl)[3]radialene (1)**. Bis(3,5-dimethylpyrazol-1yl)methane (2.00 g, 9.8 mmol) was placed in a dry two-necked flask under nitrogen. Dry tetrahydrofuran (40 mL) was added and the solution was cooled to −78 °C. *n*-Butyllithium (4.0 mL of a 2.5 M solution in hexane) was added slowly and the reaction mixture was stirred for 30 min. Tetrachlorocyclopropene (0.2 mL 1.6 mmol) was added and the solution was stirred at −78 °C for 1 h, at 0 °C for 30 min and at room temperature for 30 min. The mixture was again cooled to 0 °C and oxygen bubbled through it for 30 min at 0 °C and then for 1 h at room temperature. The resultant brown solution was quenched with water (40 mL) and extracted with dichloromethane (50 mL) followed by further dichloromethane (5 × 25 mL). The organic extracts were combined, dried over anhydrous magnesium sulfate, and the solvent was evaporated to yield a red–brown oil. Purification via alumina chromatography eluting with 9:1 CH_2_Cl_2_:MeOH, followed by silica chromatography eluting with 9:1 CHCl_3_:MeOH yielded **1** as a yellow–brown solid (150 mg, 14%). Mp 220 °C dec; ^1^H NMR (300 MHz, CDCl_3_) δ 1.80 (s, 18H, CH_3_), 2.07 (s, 18H, CH_3_), 5.69 (s, 6H, H4); ^13^C NMR (75 MHz, CDCl_3_) δ 10.6, 13.6, 102.6, 107.6, 118.2, 141.5, 150.4; HRMS (*m*/*z*): [M + H^+^] calcd for C_36_H_43_N_12_, 643.37282; found, 643.37632.

**3,3’-Diiododiphenylmethane (5)**. Based on related procedures [21,32], 3,3’-diaminodiphenylmethane (0.62 g, 3.1 mmol) in concentrated sulfuric acid (10 mL) was stirred at 0 °C. Sodium nitrite (0.62 g, 9.0 mmol) in water (6 mL) was added dropwise over a period of 10 min. The resultant solution was stirred at 0 °C for 30 min followed by the addition of potassium iodide (3.62 g, 21.8 mmol) in water (40 mL). The reaction mixture was heated at 50 °C for 1 h, then cooled to room temperature and neutralized with aqueous sodium hydroxide solution (50% w/v, 5 mL). The mixture was extracted with dichloromethane (6 × 10 mL). The organic fractions were combined and washed with 1 M hydrochloric acid solution (20 mL), 1 M sodium thiosulfate solution (20 mL), dried over anhydrous magnesium sulfate and the solvent was evaporated under vacuum to yield a brown solid. Purification via silica chromatography eluting with hexane yielded 3,3’-diiododiphenylmethane as white needles (0.73 g, 55%). Mp 63–65 °C; ^1^H NMR (300 MHz, CDCl_3_) δ 3.85 (s, 2H, CH_2_), 7.03 (t, *J* = 7.8 Hz, 2H, H5), 7.12 (d, *J* = 7.8 Hz, 2H, H6), 7.53–7.70 (overlapped d and s, 4H, H2, H4); ^13^C NMR (75 MHz, CDCl_3_) δ 40.9, 94.7, 128.2, 130.3, 135.5, 137.8, 142.6; ESIMS (*m*/*z*): [M + H^+^] 420.1.

**3,3’-Dicyanodiphenylmethane (6)**. A mixture of 3,3’-diidodiphenylmethane (0.71 g, 1.7 mmol) and copper cyanide (0.47 g, 4.1 mmol) in dry DMF (15 mL) was heated at 100 °C for 2 d. The cooled reaction mixture was diluted with ethyl acetate (40 mL) and the resultant solution was washed with concentrated ammonia solution (30 mL), water (20 mL) and brine (20 mL). Then, it was dried over anhydrous magnesium sulfate and the solvent evaporated under vacuum to yield **6** as an off-white solid (0.33 g, 90%). Mp 151–153 °C (lit. 153–154 °C [20]); ^1^H NMR (300 MHz, CDCl_3_) δ 4.05 (s, 2H, CH_2_), 7.41–7.48 (m, 6H, H2, H4, H5), 7.56 (d, *J* = 7.8 Hz, 2H, H6); ^13^C NMR (75 MHz, CDCl_3_) δ 40.8, 112.9, 118.5, 129.6, 130.5, 132.3, 133.3, 140.9; ESIMS (*m*/*z*): [M + H^+^] 218.2; FTIR v_max_/cm^−1^: 2224 (C≡N).

**Hexakis(3-cyanophenyl)[3]radialene (2)**. 3,3’-Dicyanodiphenylmethane (0.5 g, 2.28 mmol) was placed in a dry two-necked flask under argon. Dry tetrahydrofuran (20 mL) was added and the solution was cooled to −78 °C. *n*-Butyllithium (1.04 mL of a 2.2 M solution in hexane) was added slowly and the reaction mixture was stirred for 30 min. Tetrachlorocyclopropene (46 μL, 0.38 mmol) was added and the solution was stirred at −78 °C for 1 h, at 0 °C for 30 min and at room temperature for 30 min. The mixture was again cooled to 0 °C and oxygen bubbled through it for 30 min at 0 °C and then for 1 h at room temperature. The resultant brown solution was quenched with water (40 mL) and extracted with dichloromethane (30 mL) followed by further dichloromethane (5 × 10 mL). The organic extracts were combined and dried over anhydrous magnesium sulfate, then, the solvent was evaporated to yield a brown oil. Purification via silica chromatography eluting with 1:2 EtOAc/hexane yielded **2** as an orange solid (40 mg, 16%). Mp 321 °C dec; ^1^H NMR (300 MHz, CDCl_3_) δ 6.96 (s, 6H, H2), 7.23 (d, *J* = 7.9 Hz, 6H, H6), 7.30 (t, *J* = 7.9 Hz, 6H, H5), 7.59 (d, *J* = 7.9 Hz, 6H, H4); ^13^C NMR (75 MHz, CDCl_3_): δ 112.5, 117.9, 118.9, 123.0, 129.5, 132.3, 134.0, 140.6; HRMS (*m*/*z*): [M + H^+^] calcd for C_48_H_25_N_6_, 685.21352; found, 685.21196; FTIR v_max_/cm^−1^: 2228 (C≡N); Anal. calcd for C_48_H_24_N_6_·H_2_O C, 82.03; H, 3.74; N, 11.96%; found for C, 81.54; H, 4.31; N, 10.90.

**4,4’-Methyldiphthalic acid (8)**. Based on the procedure outlined [34], 4,4’-carbonyldiphthalic acid (4.4 g, 12.3 mmol) was dissolved in ethanol (100 mL). 5% Pd/C (0.88 g) was added and the resultant mixture was heated at reflux under a hydrogen atmosphere for 1 week. After cooling the mixture was filtered and the filtrate was concentrated under reduced pressure. The residue was dissolved in water (30 mL), basified with sodium hydroxide (4.0 g) and heated at reflux for 1 h. Upon cooling the solution was acidified with dilute sulfuric acid and concentrated to 30 mL under reduced pressure which resulted in the precipitation of a white solid. Filtration and subsequent drying in a desiccator overnight yielded **8** as a white solid (4.06 g, 95%). Mp 255 °C (lit. 250 °C [21]); ^1^H NMR (300 MHz, DMSO-*d*_6_) δ 4.09 (s, 2H, CH_2_), 7.39 (d, *J* = 8.0 Hz, 2H, H5), 7.81 (s, 2H, H3), 7.91 (d, *J* = 8.0 Hz, 2H, H6); ^13^C NMR (75 MHz, DMSO-*d*_6_) 130.7, 131.0, 131.4, 131.8, 134.6, 143.3, 168.0, 168.3, DMSO peak obscures CH_2_ carbon; ESIMS (-ve mode) (*m*/*z*): 343.0 ([M − H]^−^); FTIR v_max_/cm^−1^: 3435 (O–H), 1656 (C=O).

**4,4’-Methyldiphthalimide (9)** [48]. 4,4’-Methyldiphthalic acid (4.0 g, 11.6 mmol) was suspended in formamide (35 mL) and stirred at 190 °C for 2 h, then at 150 °C for 1 h, before being cooled to room temperature. The resultant precipitate was collected via filtration and washed thoroughly with water. Drying in a desiccator overnight yielded **9** as a white powder (3.01 g, 85%). Mp >280 °C. ^1^H NMR (300 MHz, DMSO-*d*_6_) δ 4.33 (s, 2H, CH_2_), 7.77–7.80 (m, 6H), 11.27 (s, 2H, NH), ^13^C NMR (75 MHz, DMSO-*d*_6_) 40.6, 123.2, 123.3, 130.8, 133.3, 134.7, 147.4, 169.0, 169.1; ESIMS (*m*/*z*): [M + H^+^] 307.4; FTIR: v_max_/cm^−1^: 3185 (N–H), 1770 and 1712 (C=O).

**4,4’-Methyldiphthalamide (10)** [48]. 4,4’-Methyldiphthalimide (2.98 g, 9.7 mmol) was finely crushed and suspended in 28% concentrated ammonia solution (40 mL). The flask was stoppered and the mixture stirred 2 d at room temperature. The resultant mixture was filtered and the precipitate was washed thoroughly with water. Drying in a desiccator overnight yielded **10** as a white powder (2.82 g, 85%). Mp > 280 °C; ^1^H NMR (300 MHz, DMSO-*d*_6_) δ 4.01 (s, 2H, CH_2_), 7.29–7.43 (m, 10H), 7.67–7.69 (m, 4H); ^13^C NMR (75 MHz, DMSO-*d*_6_) 127.9, 129.3, 133.8, 136.7, 142.0, 169.9, 170.2, DMSO peak obscures CH_2_ carbon; ESIMS (-ve mode) (*m*/*z*): 3 ([M − H^+^]^−^) 40.8; FTIR: v_max_/cm^−1^: 3329 and 3176 (N–H), 1693 and 1649 (C=O).

**4,4’-Methyldiphthalonitrile (11)**. 4,4’-Methyldiphthalamide (2.78 g, 8.2 mmol) was suspended in DMF (40 mL) and the mixture was cooled to −20 °C. Sulfonyl chloride (20 mL) was added dropwise ensuring the temperature remained below 0 °C. The resultant mixture was stirred at 0 °C for 2 h and then allowed to warm to room temperature overnight. The reaction mixture was slowly poured onto crushed ice (400 g) and stirred until the ice was completely melted. The resultant precipitate was collected via filtration, washed thoroughly with water and dried in a desiccator. Purification via silica chromatography eluting with dichloromethane yielded **11** as a white powder (1.96 g, 89%). Mp 205 °C; ^1^H NMR (300 MHz, acetone-*d*_6_) δ 4.45 (s, 2H, CH_2_), 7.92 (m, 2H), 8.04 (m, 4H); ^13^C NMR (75 MHz, acetone-*d*_6_) 41.5, 114.7, 116.4 (two signals overlapped), 116.8, 135.1, 135.4 (two signals overlapped), 146.9; ESIMS (*m*/*z*): ([M − H]^−^) 267.1; FTIR: v_max_/cm^−1^: 2233 (C≡N); Anal. calcd for C_17_H_8_N_4_ C, 76.10; H, 3.01; N, 20.89%; found C, 75.82; H, 3.01; N, 21.10.

**Hexakis(3,4-dicyanophenyl)[3]radialene (3)**. 60% Sodium hydride (50 mg, 1.26 mmol) was placed in a dry two-necked flask under argon. Degassed DMF (3 mL) was added and the suspension was stirred for 15 min. Then the mixture was cooled to 0 °C and a solution of 4,4’-methyldiphthalonitrile (322 mg, 1.2 mmol) in degassed DMF (12 mL) was added dropwise via a cannula. The resultant deep blue solution was stirred at 0 °C for 30 min and then at room temperature for 1 h. The reaction mixture was again cooled to 0 °C and tetrachlorocyclopropene (23 μL, 0.2 mmol) was added slowly. The flask was hermetically sealed and allowed to warm to room temperature; thereafter it was stirred for 2 d. The mixture was then cooled back to 0 °C and fitted with a potassium hydroxide drying tube. The solution was warmed slowly back to room temperature and stirred for a further 2 d. The deep red solution was then poured onto cold saturated ammonium chloride solution (200 mL) and stirred overnight. After dilution with water (300 mL) the mixture was filtered to yield a brown solid. Purification by silica chromatography eluting with methanol/acetone/dichloromethane 0.5:1:9 until one red spot was observed and then further eluting with acetone yielded **3** as a red solid (127 mg, 76%). Mp > 280 °C; ^1^H NMR (600 MHz, DMSO-*d*_6_/TFA) δ 7.30 (d, *J* = 7.8 Hz, 6H, H5), 7.56 (s, 6H, H3), 7.82 (d, *J* = 7.8 Hz, 6H, H6); ^13^C NMR (DMSO-*d*_6_/TFA) 114.4, 114.6, 115.5, 115.7, 122.1, 122.4, 133. 7, 135.3, 135.6, 143.7; HRMS (-ve mode) (*m*/*z*): [M]^−^ calcd for C_54_H_18_N_12_, 834.17829; found, 834.17629; FTIR: v_max_/cm^−1^: 2235 (C≡N); Anal. calcd for C_54_H_18_N_12_·H_2_O C, 76.04; H, 2.37; N, 19.71%; found, C, 75.67; H, 2.37; N, 19.21.

## Supporting Information

File 1 ^1^H and ^13^C NMR spectra of all compounds, cyclic voltammograms of hexaaryl[3]radialenes, crystal data and structure refinement for **3**.

File 2 Crystallographic information file for acetonitrile solvate of hexakis(3,4-dicyanophenyl)[3]radialene.
