# Supplementary material for: Fluorescent hexaaryl- and hexa-heteroaryl[3]radialenes: Synthesis, structures, and properties
Source: Beilstein J Org Chem. 2012 Jan 11;8:71–80. doi: 10.3762/bjoc.8.7 (PMC3302100; doi:10.3762/bjoc.8.7)
Supplement: File 1 — 1H and 13C NMR spectra of all compounds, cyclic voltammograms of hexaaryl[3]radialenes, crystal data and structure refinement for 3. [file Beilstein_J_Org_Chem-08-71-s001.pdf]

Supporting Information File 1

for

Fluorescent hexaaryl- and hexa-heteroaryl[3]radialenes: Synthesis, structures, and properties

Antonio Avellaneda, Courtney A. Hollis, Xin He and Christopher J. Sumby*

Address: School of Chemistry & Physics, The University of Adelaide, Adelaide, SA 5005, Australia. Phone: +61 8 8303 7406. Fax: +61 8 8303 4358

Email: Christopher J. Sumby - christopher.sumby@adelaide.edu.au

* Corresponding author

Table of contents

^{1}H and ^{13}C NMR spectra of all compounds **S2–S10**

Cyclic voltammograms of hexaaryl[3]radialenes **S11–S12**

Crystal data and structure refinement for 3 **S13**

^{1}H and ^{13}C NMR spectra of all compounds

Solvent peaks are marked with an asterisk.

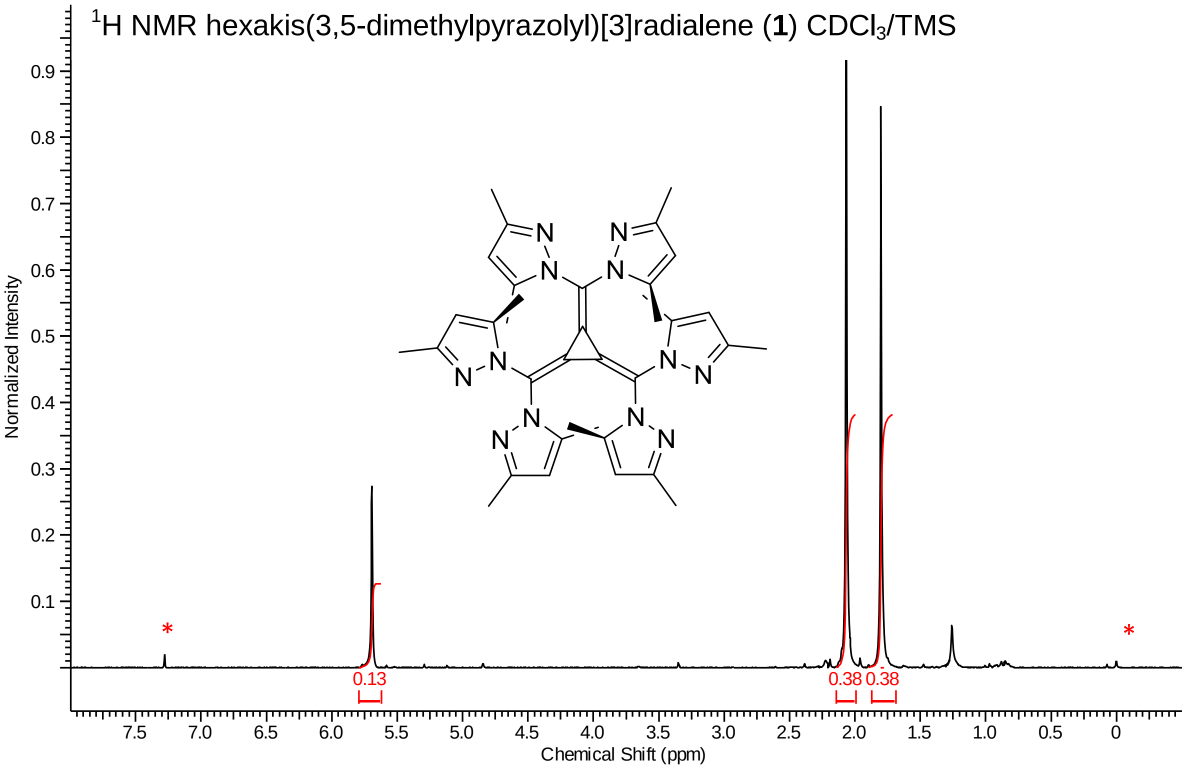

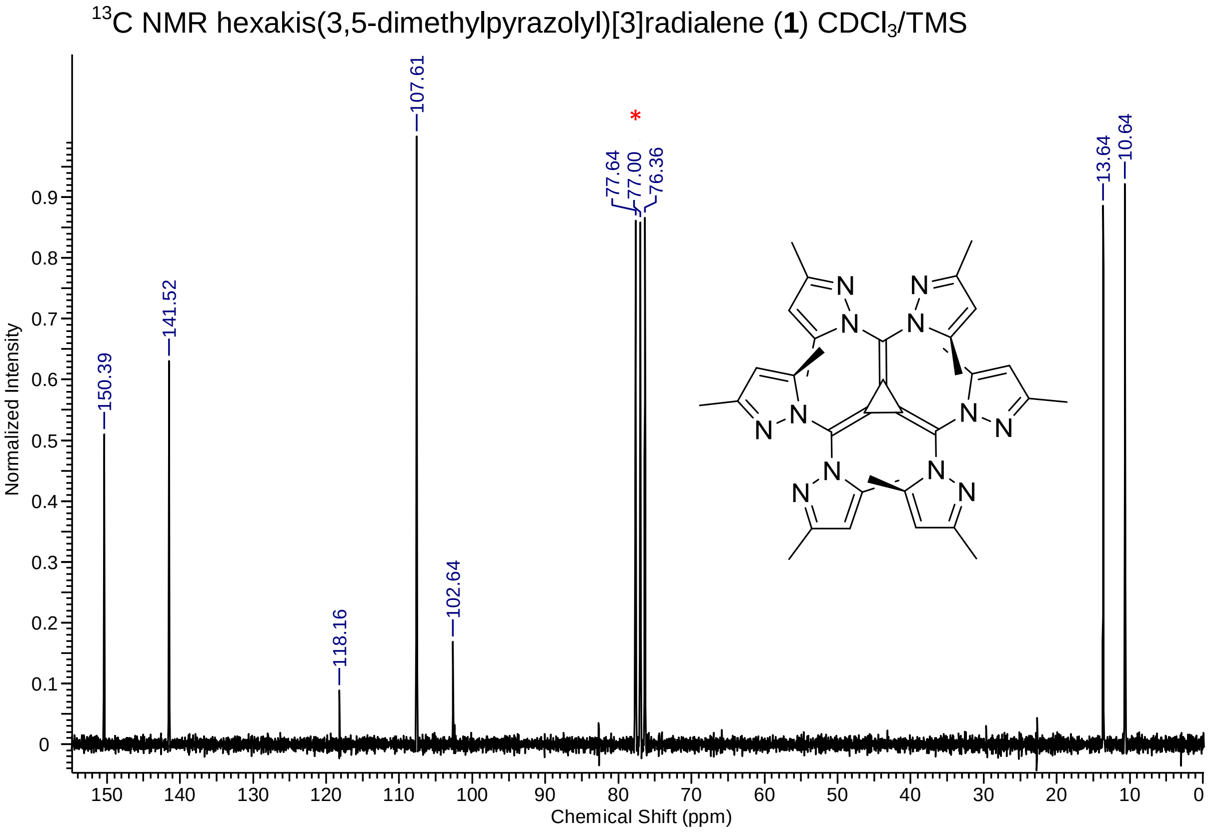

^{1}H NMR 3,3'-diiododiphenylmethane (**5**) $CDCl_3$/TMS

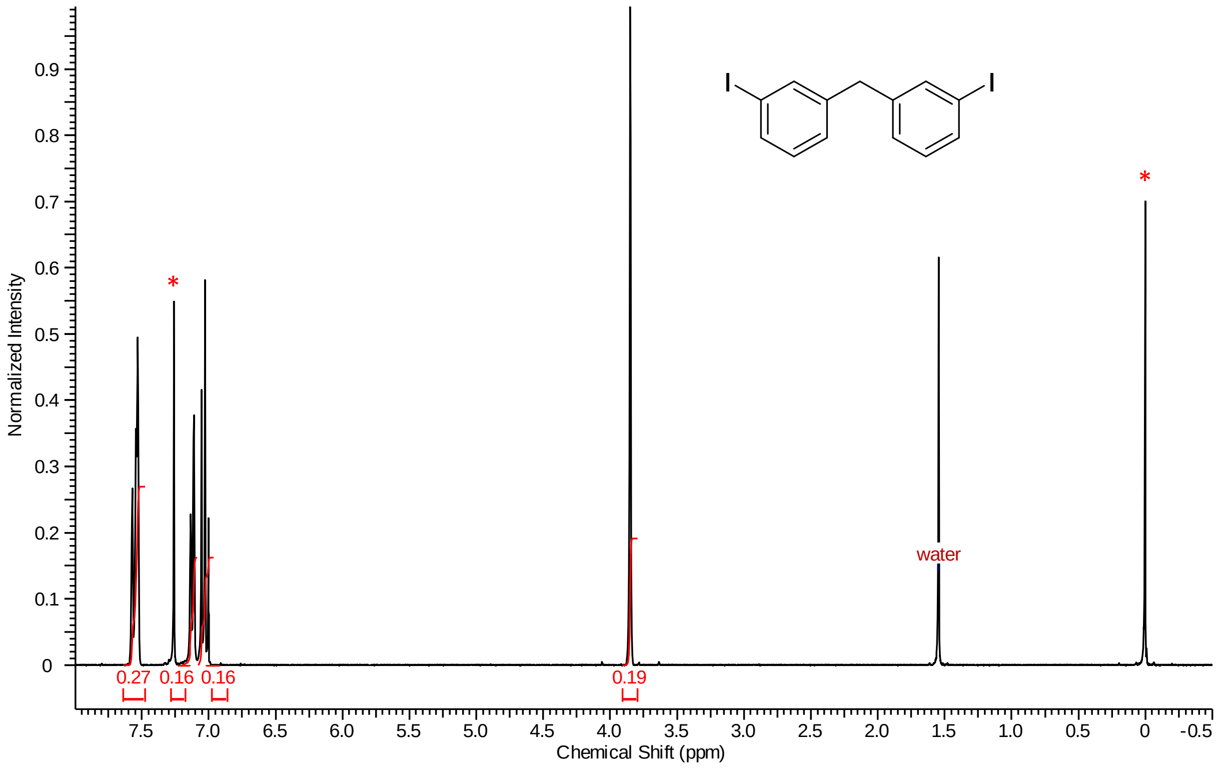

^{13}C NMR 3,3'-diiododiphenylmethane (**5**) $CDCl_3$/TMS

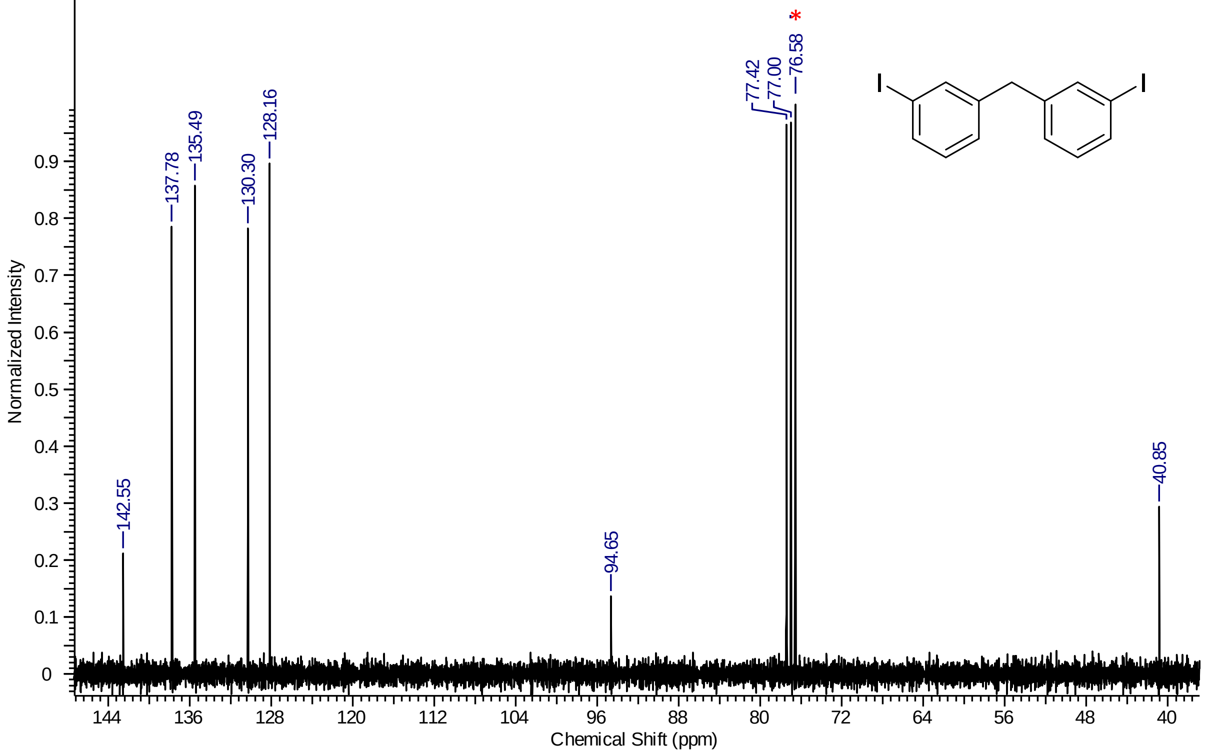

^{1}H NMR 3,3'-dicyanodiphenylmethane (**6**) $CDCl_3$/TMS

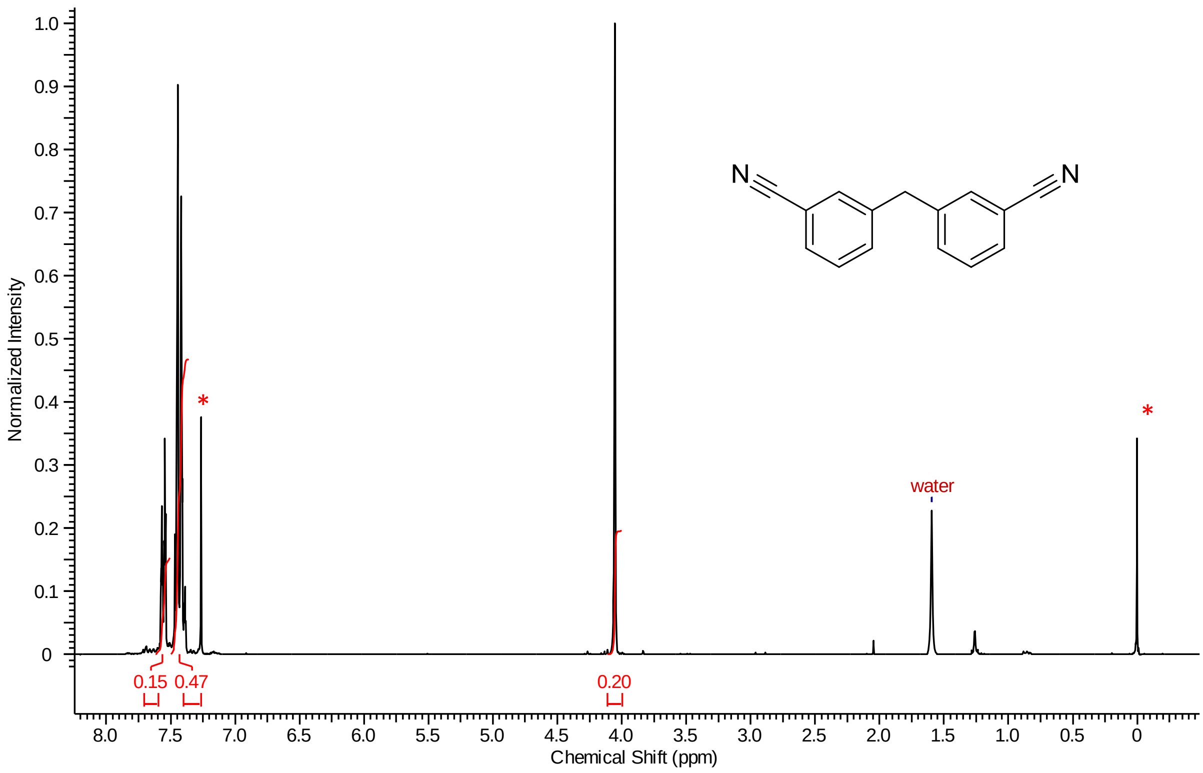

^{13}C NMR 3,3'-dicyanodiphenylmethane (**6**) $CDCl_3$/TMS

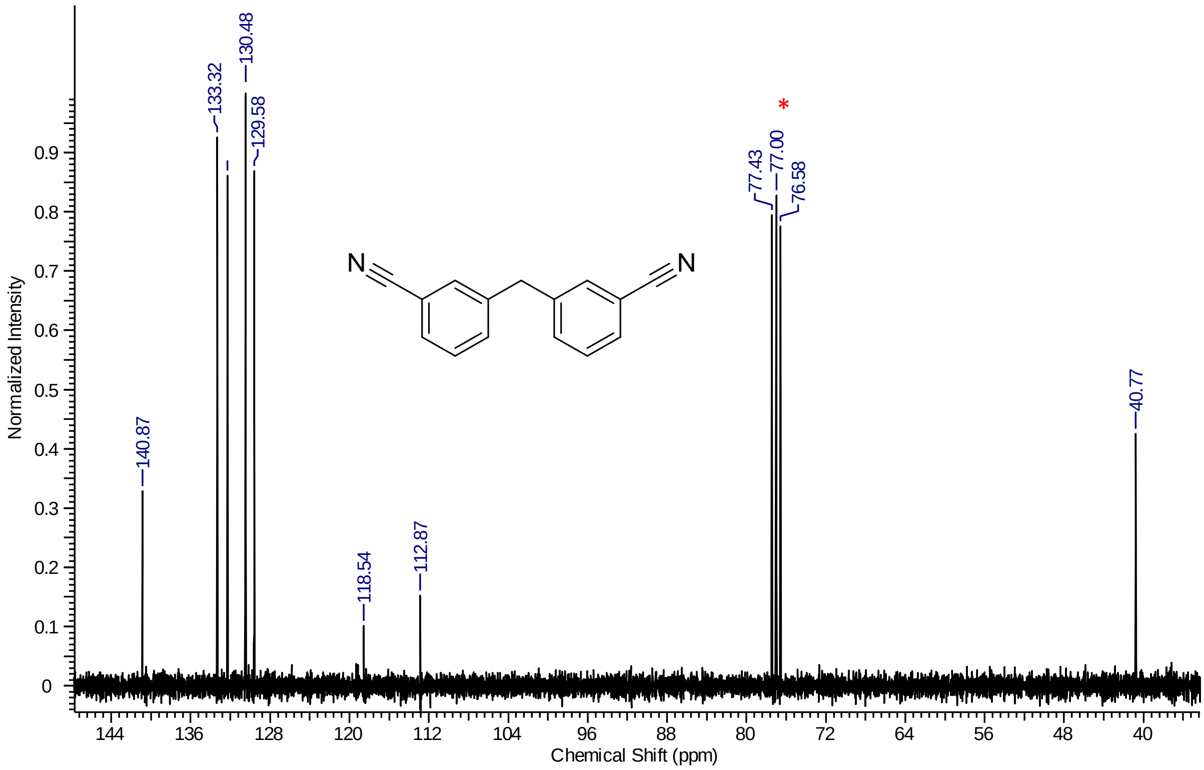

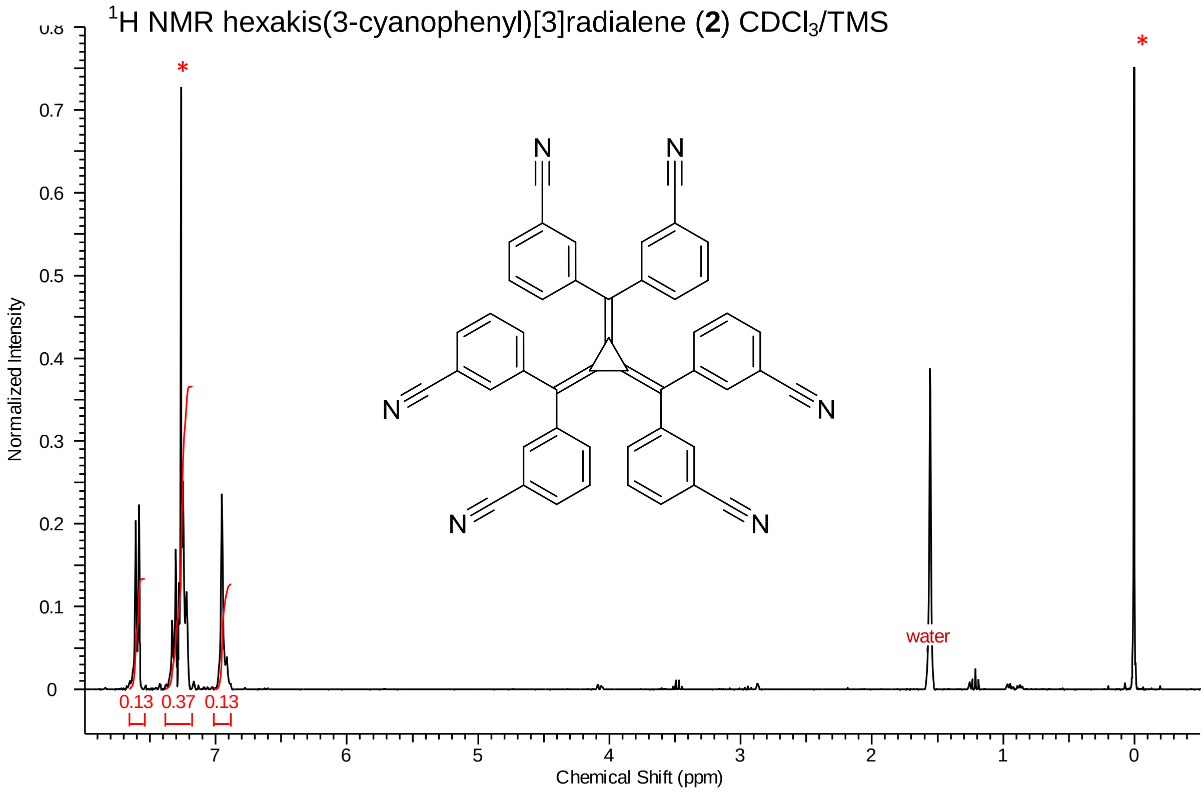

¹H NMR hexakis(3-cyanophenyl)[3]radialene (2) CDCl3/TMS
Normalized Intensity
Chemical Shift (ppm)
0.13
0.37
0.13
water

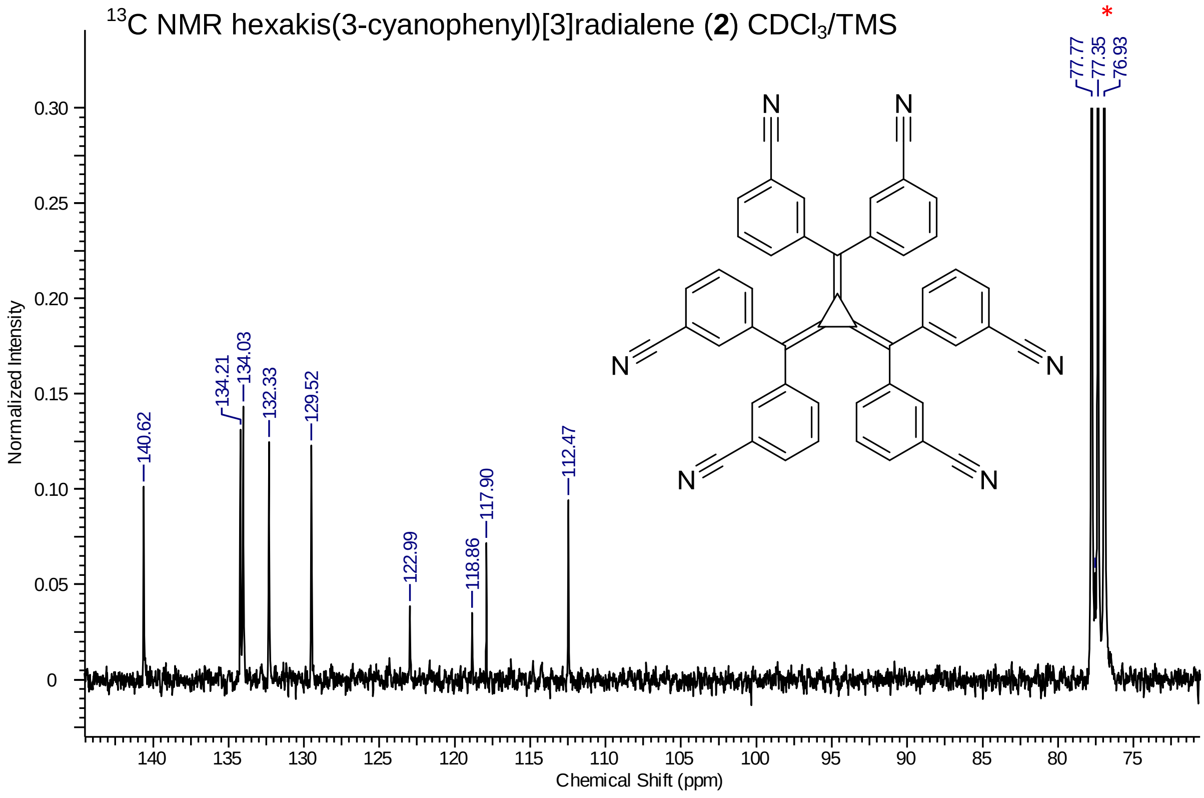

¹³C NMR hexakis(3-cyanophenyl)[3]radialene (2) CDCl3/TMS
Normalized Intensity
Chemical Shift (ppm)
140.62
134.21
134.03
132.33
129.52
122.99
118.86
117.90
112.47
77.77
77.35
76.93

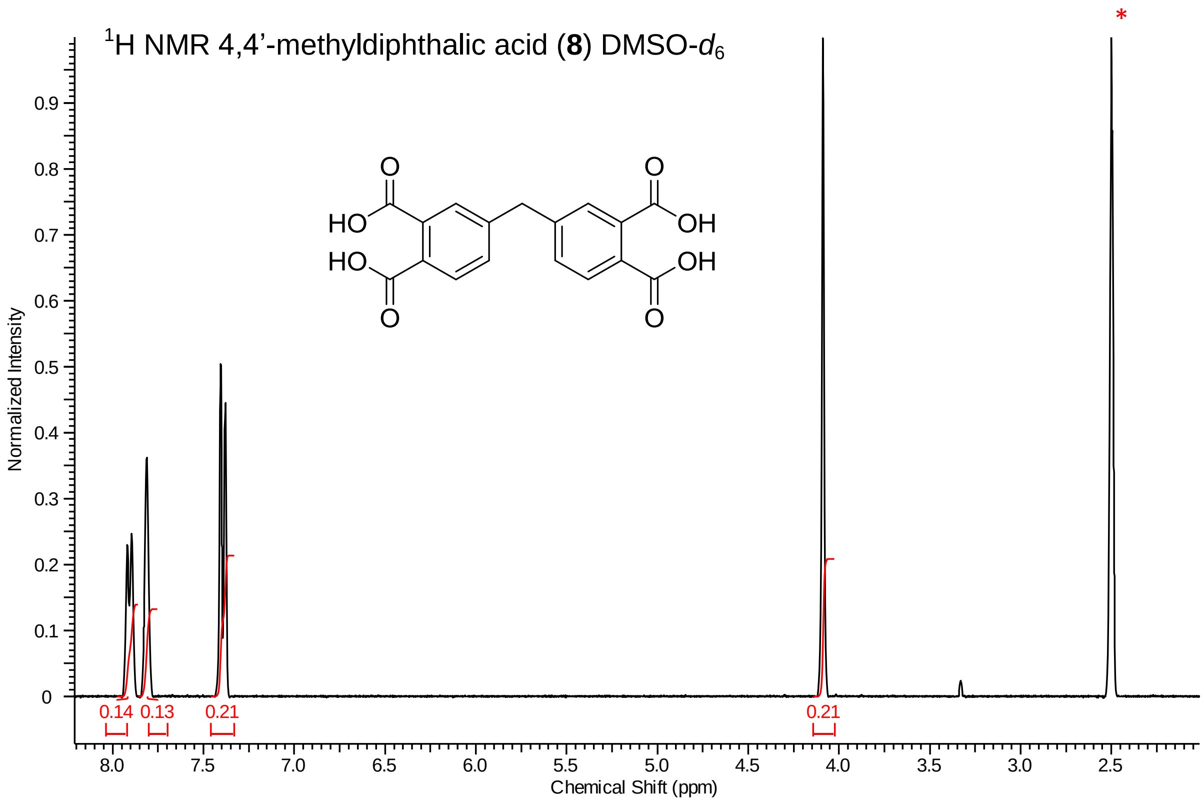

^{1}H NMR 4,4'-methyldiphthalic acid (**8**) DMSO-d_6
Normalized Intensity
Chemical Shift (ppm)
0.14
0.13
0.21
0.21
HO
HO
OH
OH
O
O
O
O

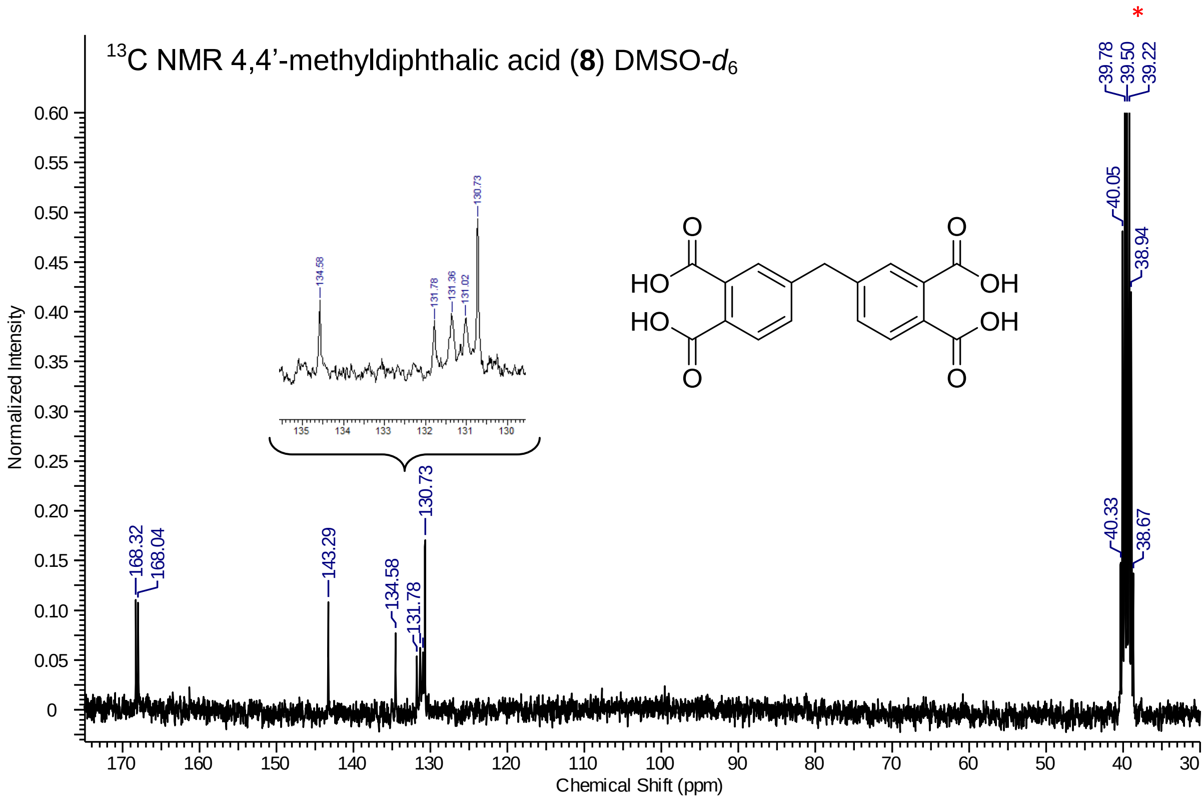

^{13}C NMR 4,4'-methyldiphthalic acid (**8**) DMSO-d_6
Normalized Intensity
Chemical Shift (ppm)
168.32
168.04
143.29
134.58
131.78
130.73
131.36
131.02
40.33
40.05
39.78
39.50
39.22
38.94
38.67
HO
HO
OH
OH
O
O
O
O

^{1}H NMR 4,4'-methyldiphthalimide (**9**) DMSO-d_6

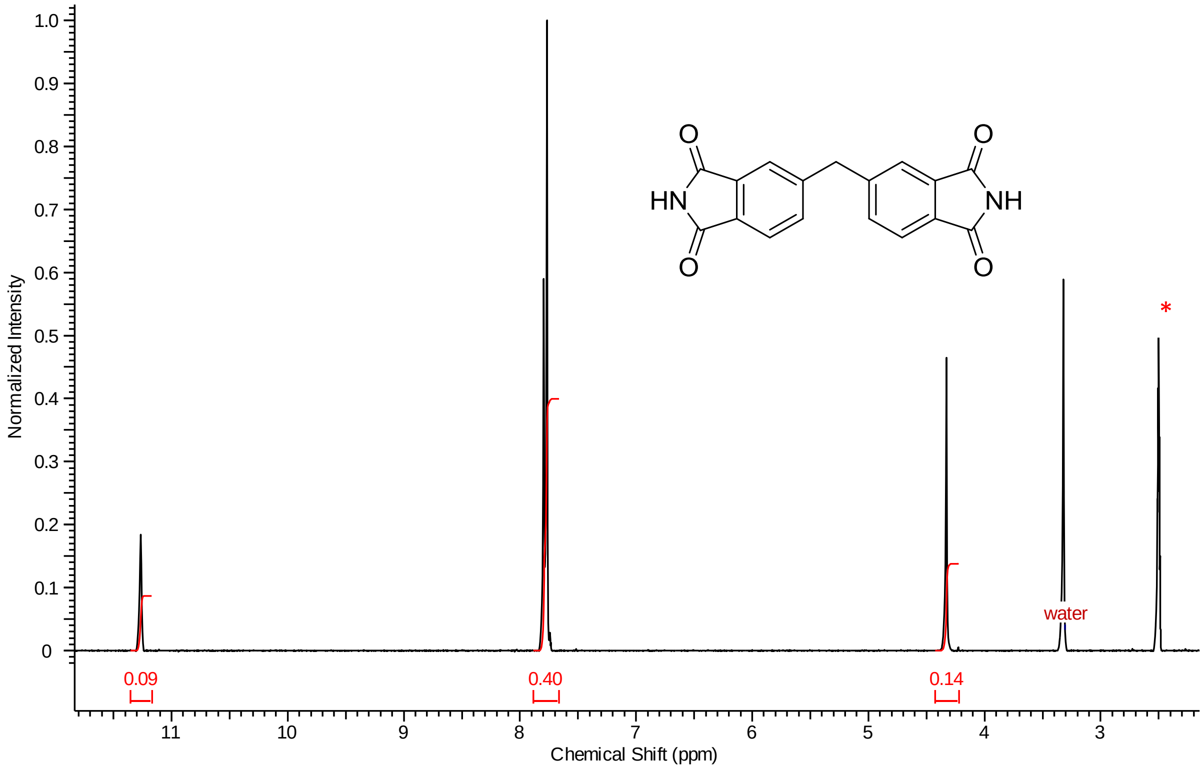

^{13}C NMR 4,4'-methyldiphthalimide (**9**) DMSO-d_6

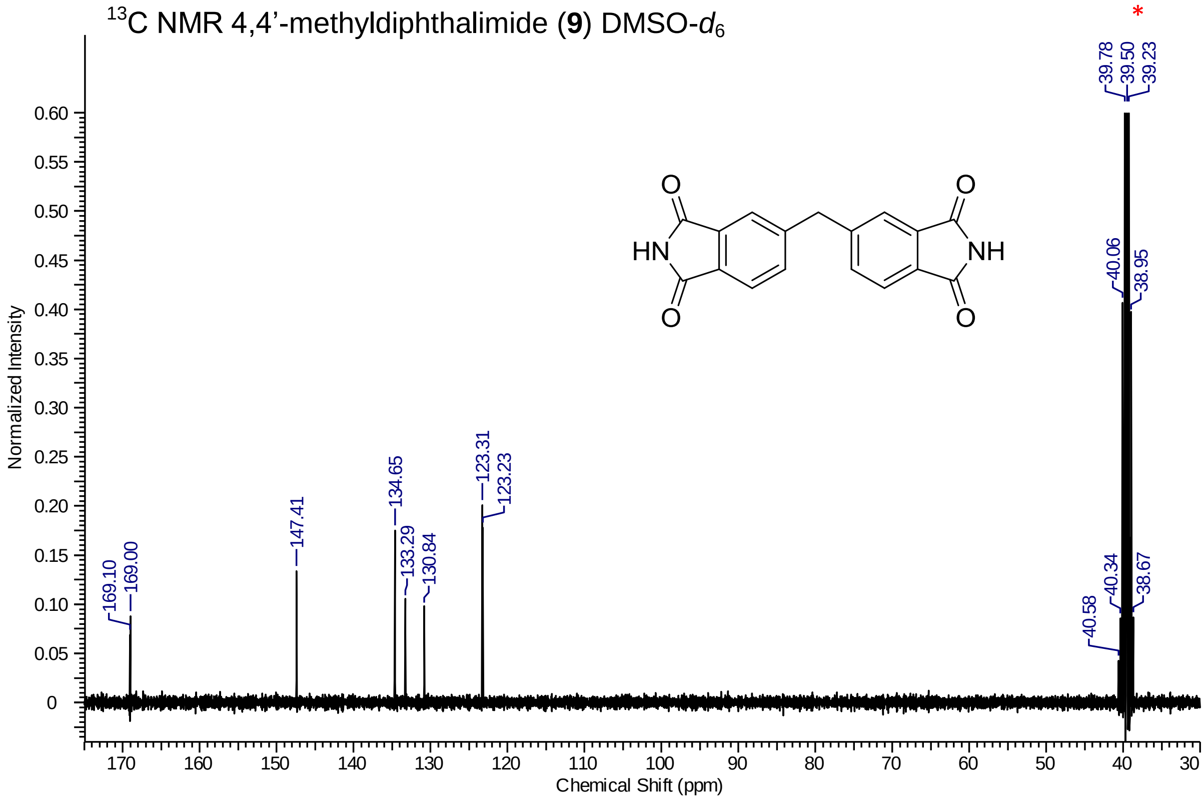

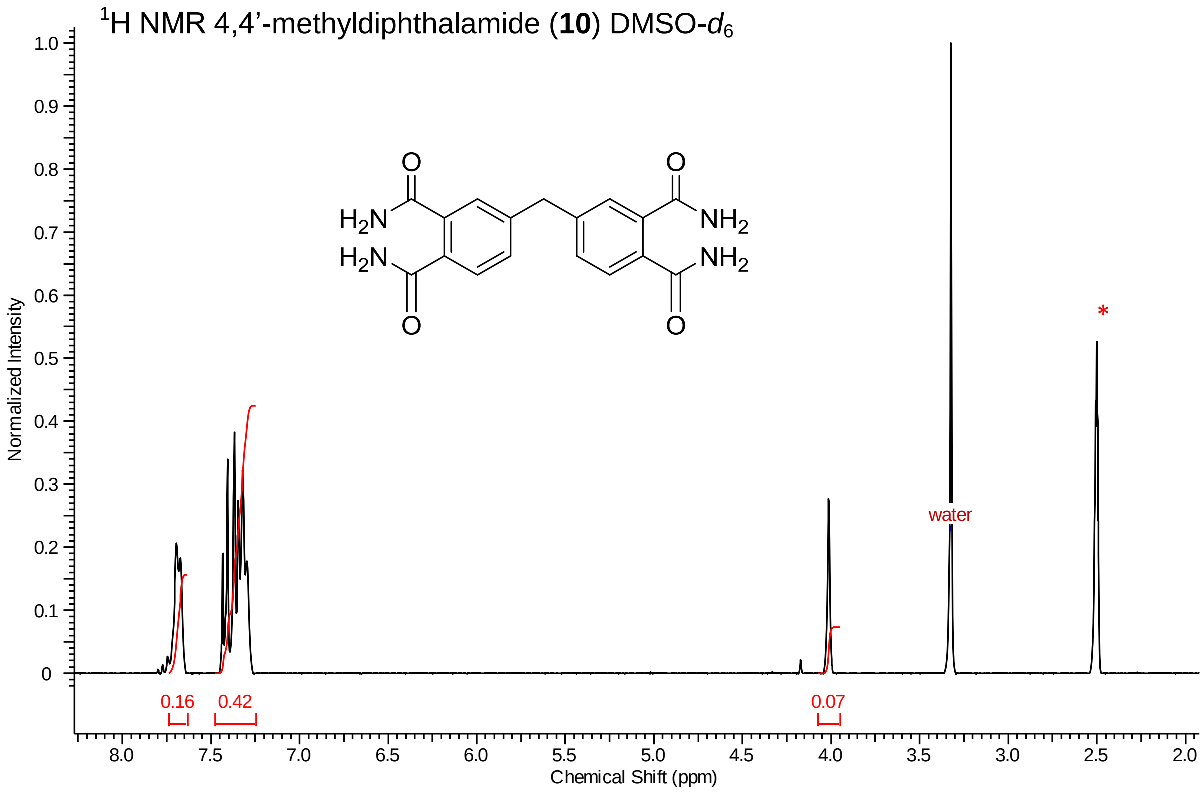

1H NMR 4,4'-methyldiphthalamide (10) DMSO-d6
H2N
H2N
O
O
NH2
NH2
O
O
Normalized Intensity
1.0
0.9
0.8
0.7
0.6
0.5
0.4
0.3
0.2
0.1
0
*
water
0.16
0.42
0.07
8.0
7.5
7.0
6.5
6.0
5.5
5.0
4.5
4.0
3.5
3.0
2.5
2.0
Chemical Shift (ppm)

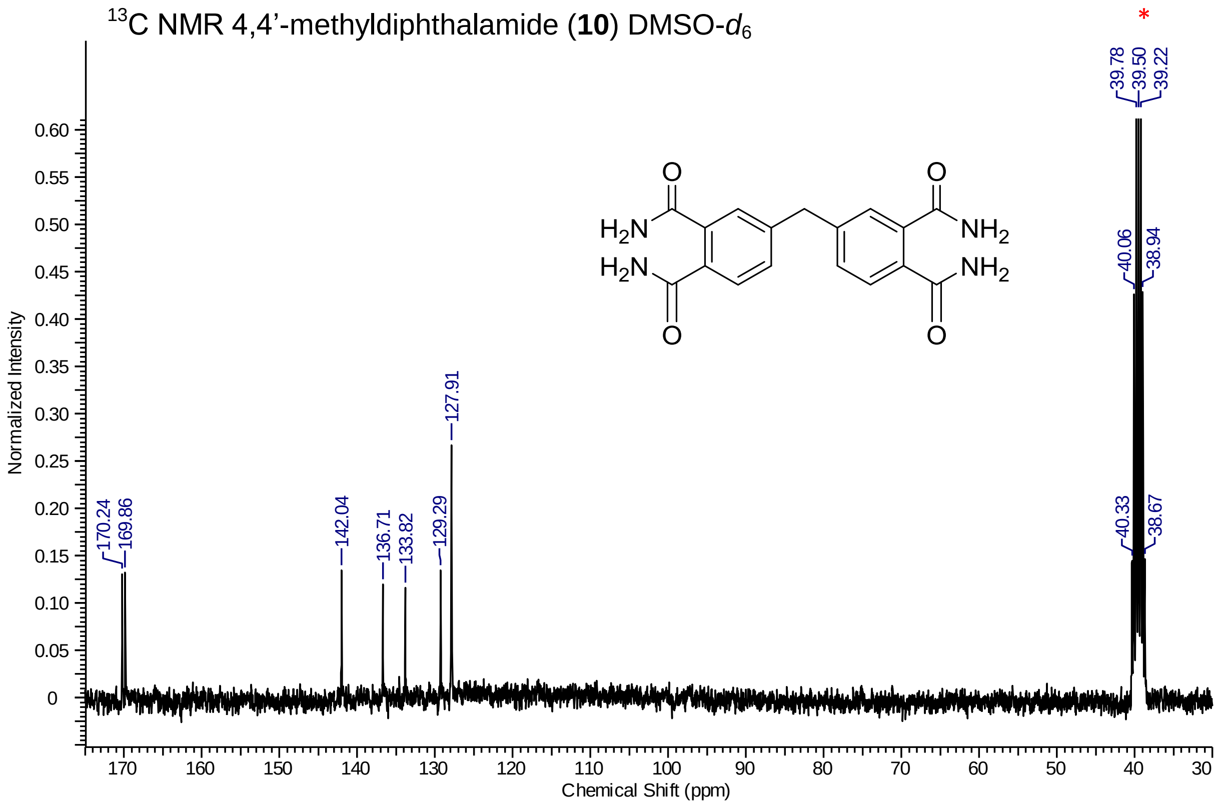

13C NMR 4,4'-methyldiphthalamide (10) DMSO-d6
*
39.78
39.50
39.22
40.06
38.94
40.33
38.67
170.24
169.86
142.04
136.71
133.82
129.29
127.91
H2N
H2N
O
O
NH2
NH2
O
O
Normalized Intensity
0.60
0.55
0.50
0.45
0.40
0.35
0.30
0.25
0.20
0.15
0.10
0.05
0
170
160
150
140
130
120
110
100
90
80
70
60
50
40
30
Chemical Shift (ppm)

^{1}H NMR 4,4'-methyldiphthalonitrile (**11**) acetone-d_6

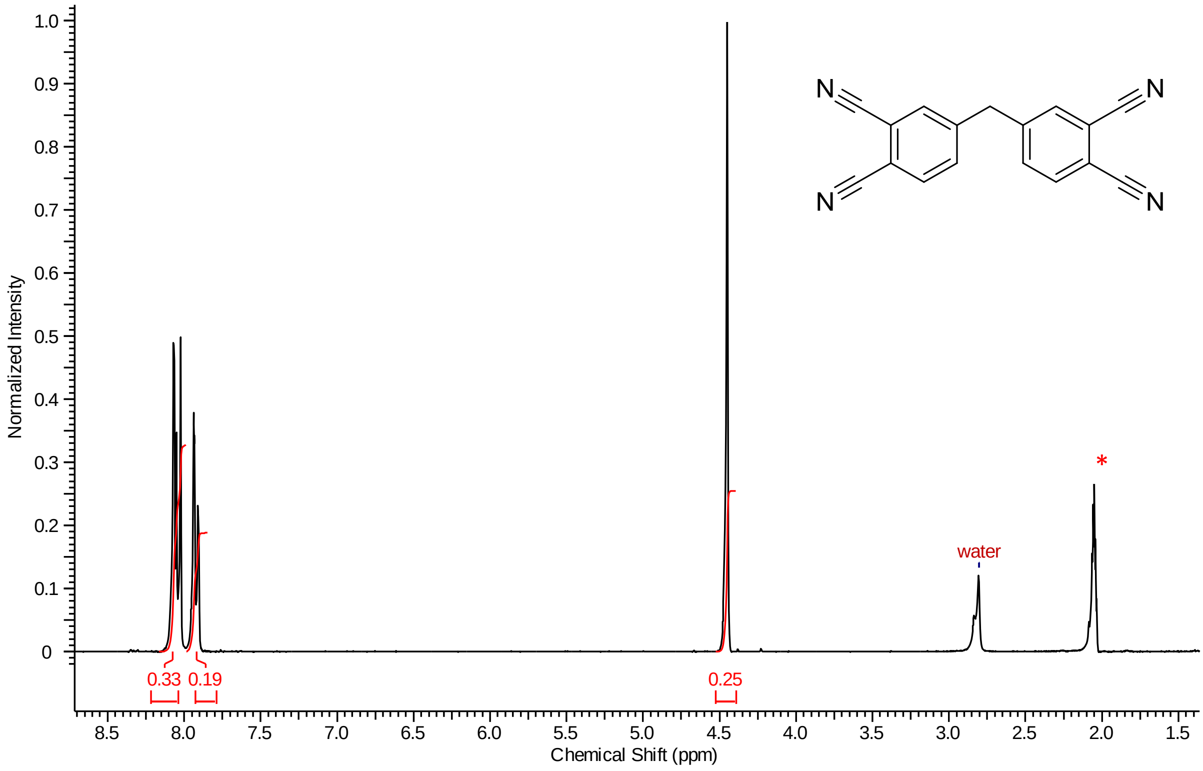

^{13}C NMR 4,4'-methyldiphthalonitrile (**11**) acetone-d_6

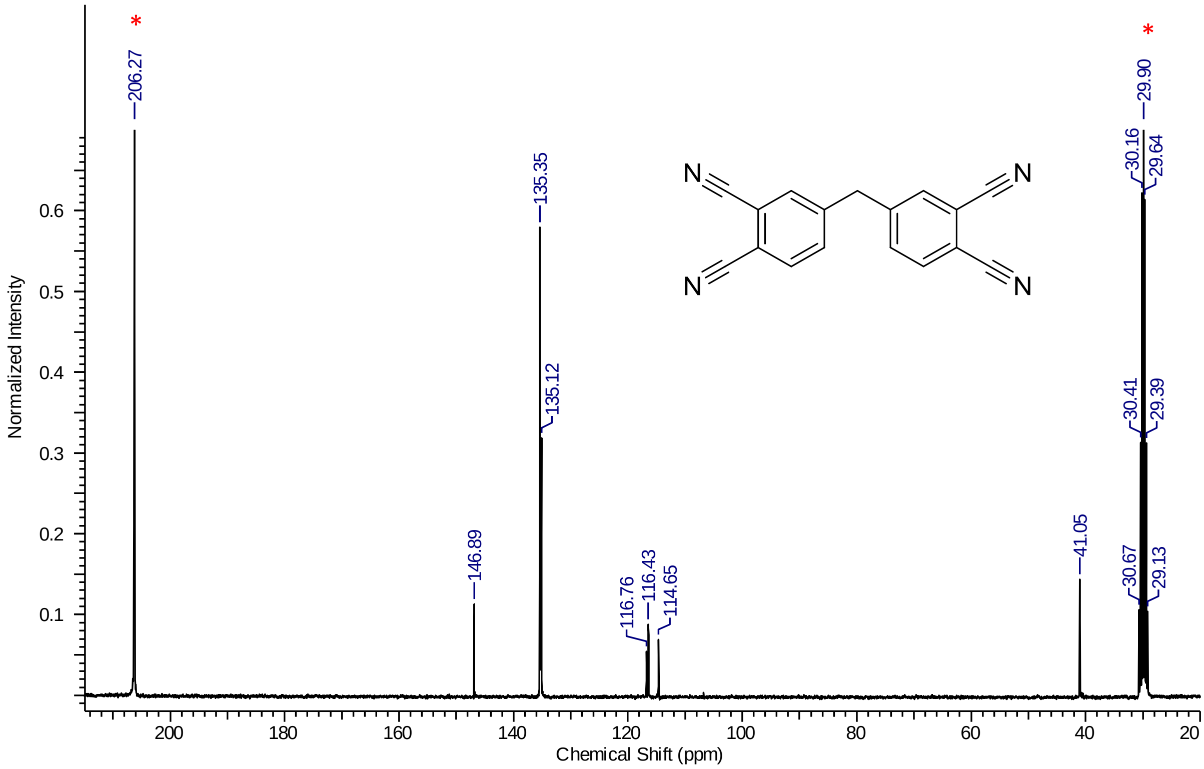

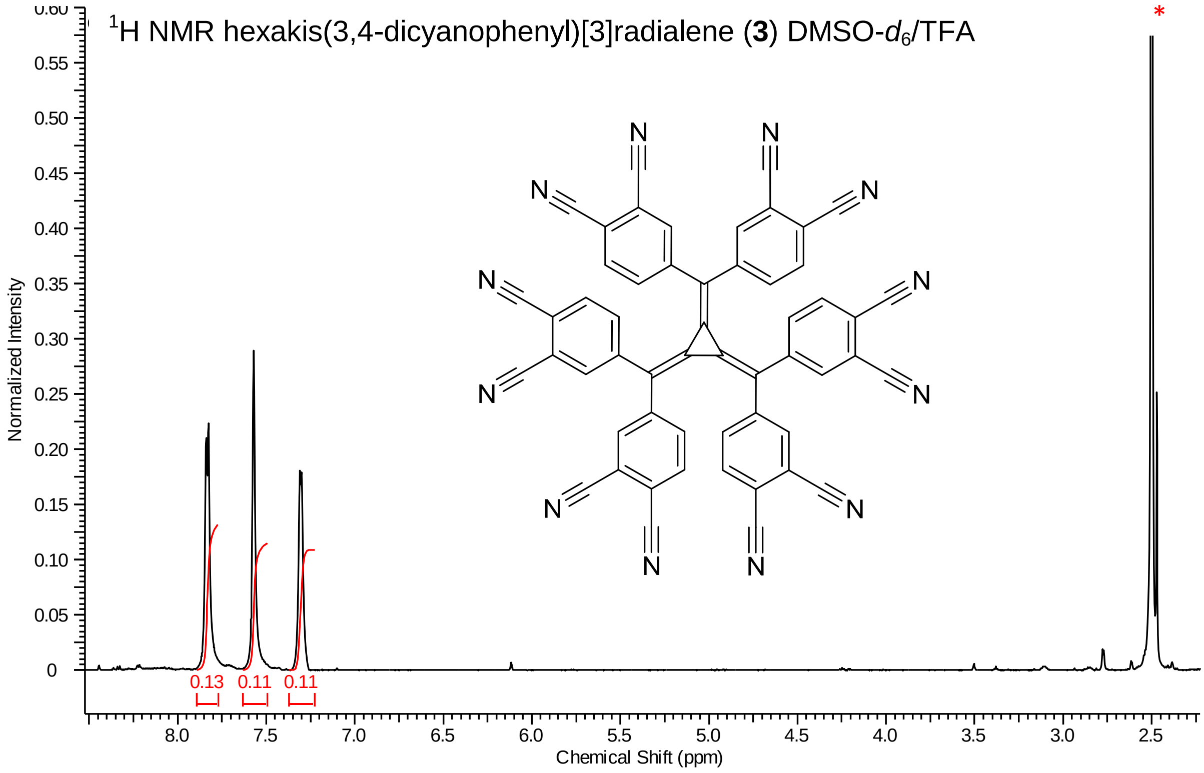

1H NMR hexakis(3,4-dicyanophenyl)[3]radialene (3) DMSO-d6/TFA
Normalized Intensity
Chemical Shift (ppm)
0.13
0.11
0.11

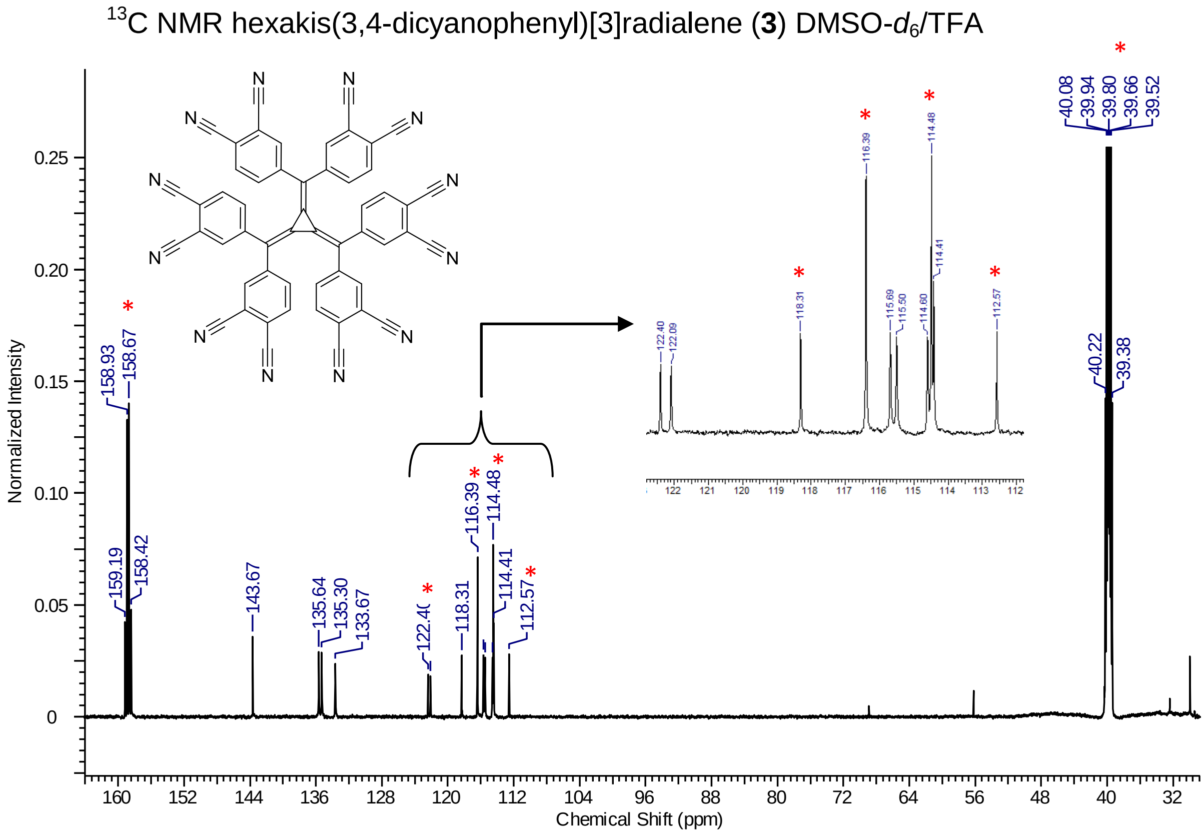

13C NMR hexakis(3,4-dicyanophenyl)[3]radialene (3) DMSO-d6/TFA
Normalized Intensity
Chemical Shift (ppm)
158.93
158.67
159.19
158.42
143.67
135.64
135.30
133.67
122.40
118.31
116.39
114.48
114.41
112.57
40.08
39.94
39.80
39.66
39.52
40.22
39.38

Cyclic voltammograms of hexaaryl[3]radialenes

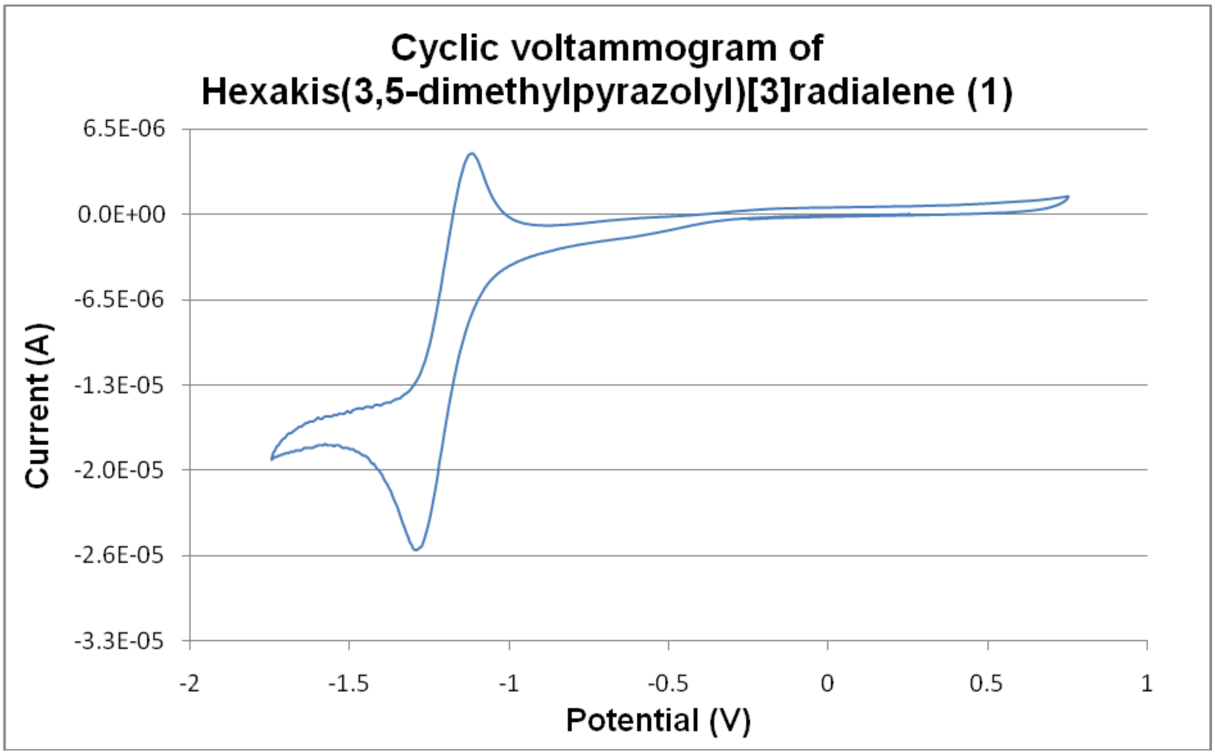

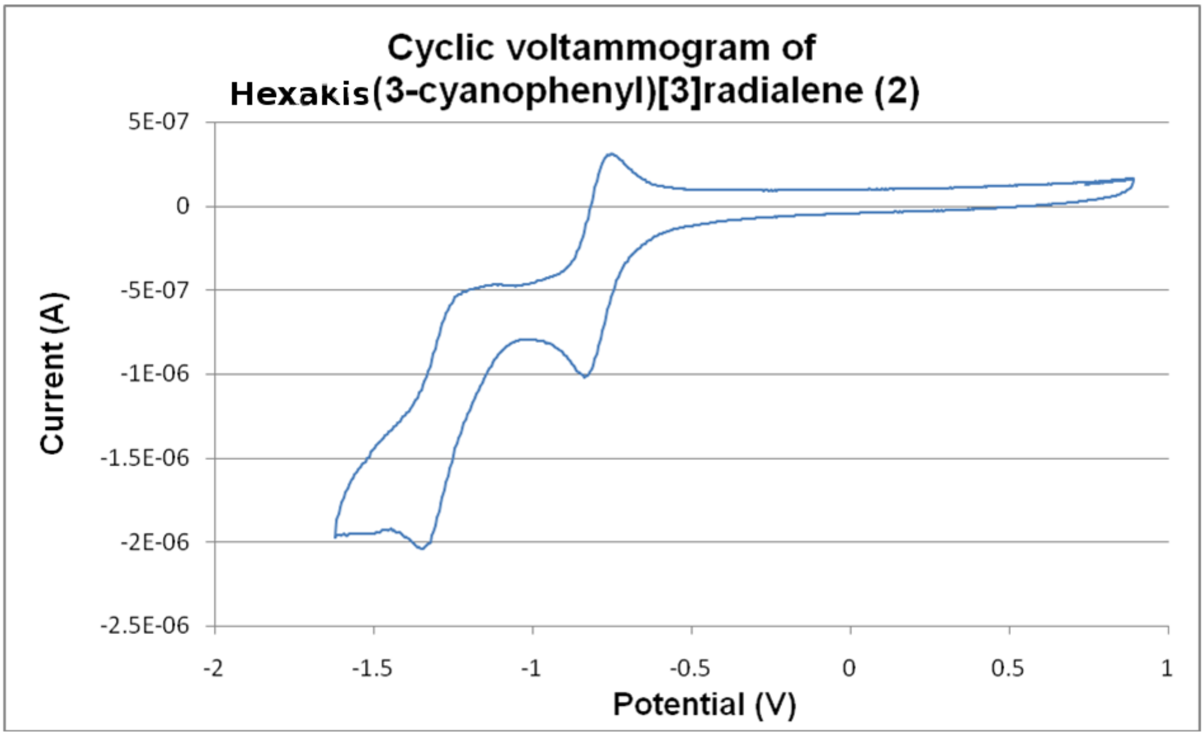

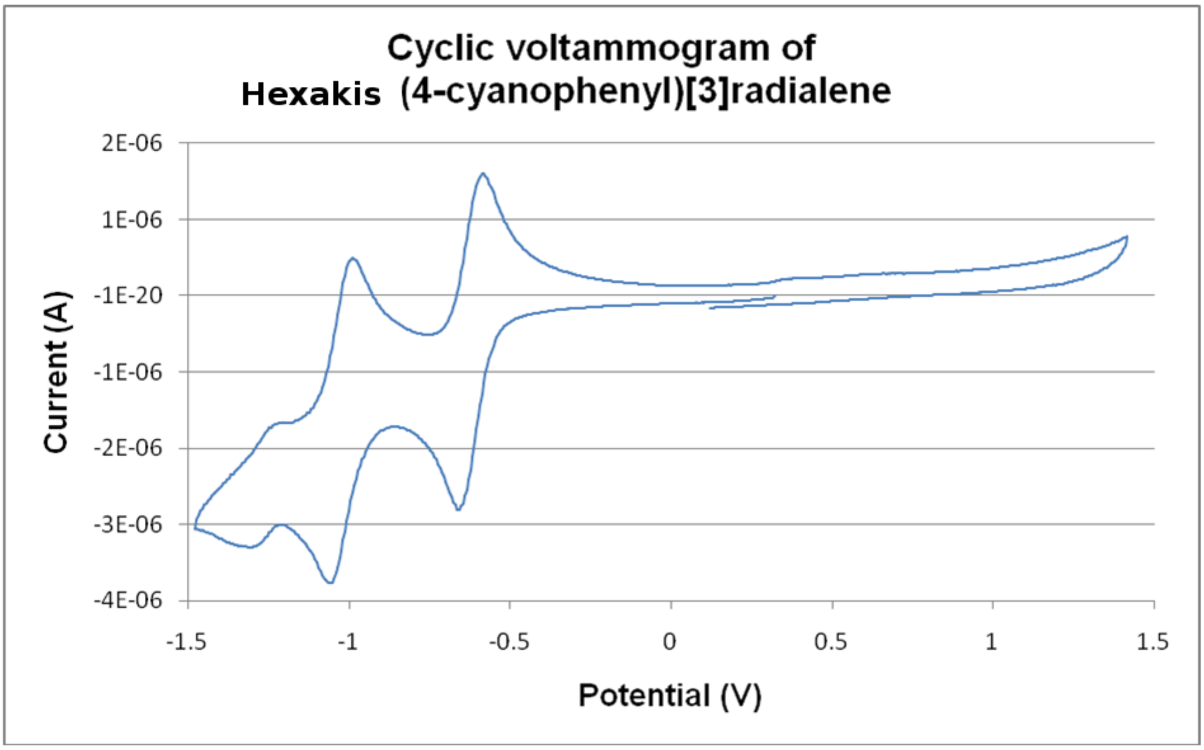
Cyclic voltammogram of
Hexakis (4-cyanophenyl)[3]radialene
2E-06
1E-06
-1E-20
-1E-06
-2E-06
-3E-06
-4E-06
Current (A)
-1.5
-1
-0.5
0
0.5
1
1.5
Potential (V)

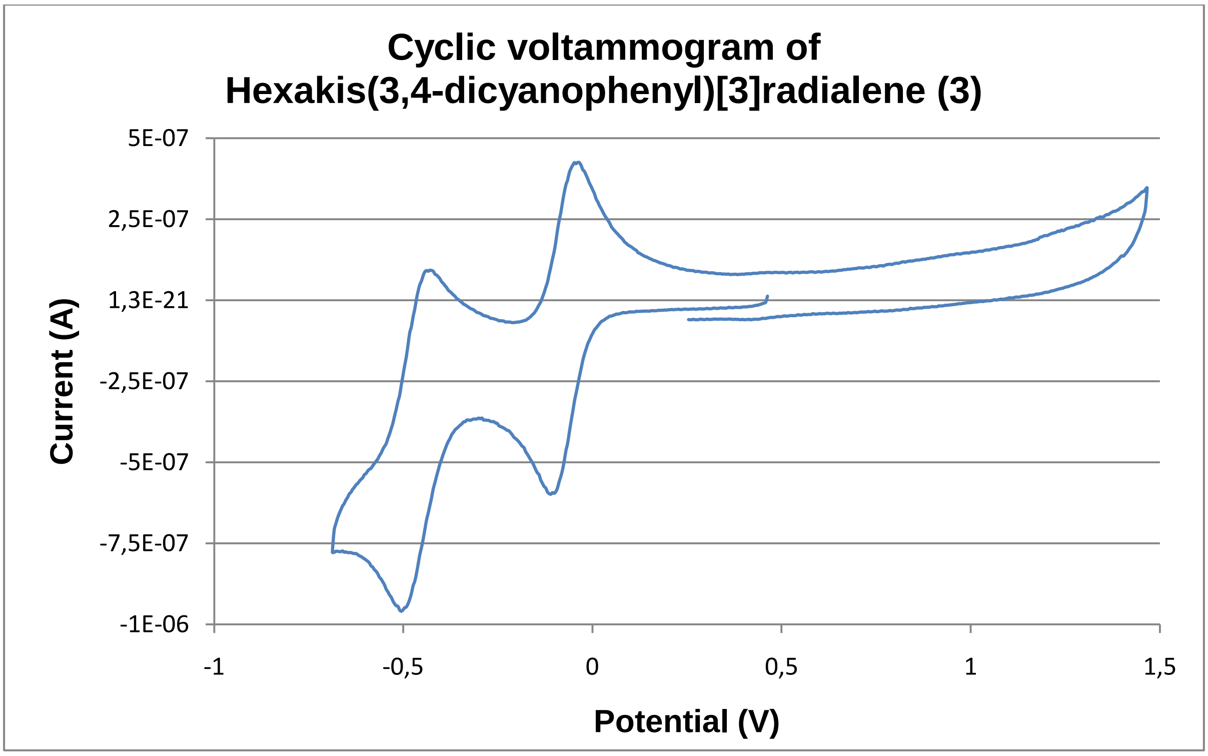
Cyclic voltammogram of
Hexakis(3,4-dicyanophenyl)[3]radialene (3)
5E-07
2,5E-07
1,3E-21
-2,5E-07
-5E-07
-7,5E-07
-1E-06
Current (A)
-1
-0,5
0
0,5
1
1,5
Potential (V)

Table 1: Crystal data and structure refinement for 3.

Empirical formula	$C_{56}H_{21}N_{13}$	
Formula weight	875.86	
Temperature	150(2) K	
Wavelength	0.7107 Å	
Crystal system	Monoclinic	
Space group	$P2_1/c$	
Unit cell dimensions	a = 13.7345(13) Å	α = 90°
	b = 12.6611(9) Å	β = 93.942(8)°
	c = 25.128(2) Å	γ = 90°
Volume	4359.3(6) Å3	
Z	4	
Density (calculated)	1.335 Mg/m^3	
Absorption coefficient	0.084 mm^{-1}	
F(000)	1792	
Crystal size	0.150 × 0.128 × 0.040 mm^3	
Theta range for data collection	2.67 to 23.26°	
Index ranges	−15<=h<=15, −14<=k<=14, −27<=l<=27	
Reflections collected	28680	
Independent reflections	6241 [R_{int} = 0.1149]	
Completeness to theta = 23.26°	99.9%	
Absorption correction	Semi-empirical from equivalents	
Max. and min. transmission	1.00000 and 0.84680	
Refinement method	Full-matrix least-squares on F2	
Data / restraints / parameters	6241/0/623	
Goodness-of-fit on F2	1.025	
Final R indices [I>2sigma(I)]	R_1 = 0.0682, wR_2 = 0.1296	
R indices (all data)	R_1 = 0.1324, wR_2 = 0.1608	
Largest diff. peak and hole	0.362 and −0.320 e.Å^{-3}	
